# Comparing Badger (*Meles meles*) Management Strategies for Reducing Tuberculosis Incidence in Cattle

**DOI:** 10.1371/journal.pone.0039250

**Published:** 2012-06-27

**Authors:** Graham C. Smith, Robbie A. McDonald, David Wilkinson

**Affiliations:** 1 The Food and Environment Research Agency, York, North Yorkshire, United Kingdom; 2 Environment and Sustainability Institute, University of Exeter, Penryn, Cornwall, United Kingdom; University of California, Berkeley, United States of America

## Abstract

Bovine tuberculosis (bTB), caused by *Mycobacterium bovis*, continues to be a serious economic problem for the British cattle industry. The Eurasian badger (*Meles meles*) is partly responsible for maintenance of the disease and its transmission to cattle. Previous attempts to manage the disease by culling badgers have been hampered by social perturbation, which in some situations is associated with increases in the cattle herd incidence of bTB. Following the licensing of an injectable vaccine, we consider the relative merits of management strategies to reduce bTB in badgers, and thereby reduce cattle herd incidence. We used an established simulation model of the badger-cattle-TB system and investigated four proposed strategies: business as usual with no badger management, large-scale proactive badger culling, badger vaccination, and culling with a ring of vaccination around it. For ease of comparison with empirical data, model treatments were applied over 150 km^2^ and were evaluated over the whole of a 300 km^2^ area, comprising the core treatment area and a ring of approximately 2 km. The effects of treatment were evaluated over a 10-year period comprising treatment for five years and the subsequent five year period without treatment. Against a background of existing disease control measures, where 144 cattle herd incidents might be expected over 10 years, badger culling prevented 26 cattle herd incidents while vaccination prevented 16. Culling in the core 150 km^2^ plus vaccination in a ring around it prevented about 40 cattle herd breakdowns by partly mitigating the negative effects of culling, although this approach clearly required greater effort. While model outcomes were robust to uncertainty in parameter estimates, the outcomes of culling were sensitive to low rates of land access for culling, low culling efficacy, and the early cessation of a culling strategy, all of which were likely to lead to an overall increase in cattle disease.

## Introduction

Bovine tuberculosis (bTB), caused by *Mycobacterium bovis*, continues to be a serious economic problem for the British cattle industry. Eurasian badgers (*Meles meles*) are a significant reservoir of bTB in large parts of England, Wales and Ireland [1], and a large-scale field trial in England (the Randomised Badger Culling Trial, RBCT) gave conclusive evidence that they are responsible for a significant proportion of transmission of the disease to cattle [2]. However, this trial also indicated that, although proactive culling of badgers significantly reduced the number of Cattle Herd Breakdowns (CHBs) within the culling area, in the adjoining land the CHB rate increased significantly for a period [3]. Thus, the net benefit of culling was somewhat diminished by a temporary detriment experienced in the periphery of the culling area [4]. The most likely reason for the observed increase in the adjoining land was the apparent perturbation of badger social structure [3]. This perturbation effect is where badger culling causes a breakdown of the badger's territorial social system, increasing badger movements and, it is hypothesised, increases contact rates and disease spread [5], [6].

Following completion of a clinical evaluation of the use of Bacillus Calmette-Guerin (BCG) as a vaccine for badgers [7], and the granting of a limited marketing authorisation for the use of injected BadgerBCG in badgers in 2010 [8], the government in England has considered badger control strategies to reduce the CHB rate in those parts of England where bTB is worst. Here, we have used computer modelling to compare three possible badger control strategies, as identified in the Department of Environment, Food and Rural Affairs (Defra) public consultation [9]: (1) culling, (2) vaccination, and (3) a combined strategy of vaccination in a ring around a culling area (hereafter referred to as “culling plus ring vaccination"). The main objective was to compare the disease outcomes of the suggested badger management strategies, relative to a “business as usual" approach. Culling plus ring vaccination was proposed specifically so that it might mitigate the negative effects of badger culling (i.e. social perturbation at the edge of a culled area). To this end we utilised and adapted an existing computer model that has already been used to examine several approaches to badger/cattle bTB management for Defra and the Welsh Government.

## Methods

The model used for this study was a modification of the Badger-Cattle model used previously to model bovine tuberculosis infection [10], [11], [12], [13], [14], [15], [16]. It is an individual-based spatial stochastic badger/bTB model combined with a cattle layer so that spatially realistic interactions and bTB transmission between badgers and cattle can be simulated. We used a model time step of 2 months so that farm management (such as repeat bTB tests) could be simulated. The badger and cattle layers were both modelled on a grid of 100×100 cells, each representing 200×200 m (total grid area represented 400 km^2^). The grid was wrapped to form a torus to eliminate edge effects. Model parameter settings (e.g. badger and farm density) were based on means from an area of six counties in the South West of England (Avon, Cornwall, Devon, Gloucestershire, Hereford & Worcester, and Wiltshire) that comprises mixed farmland with high densities of badgers and cattle and a high CHB rate.

**Table 1:** Effects of culling, vaccination, and culling plus ring vaccination on the mean number of infected badgers per social group.

(A) during	No badger control	Badger culling	Badger vaccination	Badger culling & ring vaccination
Core plus Ring	1.30	1.16 (−10%)	1.10 (−15%)	0.89 (−31%)
Core Area	1.32*	0.71 (−46%)	0.95 (−28%)	0.64 (−51%)
Ring Area	1.27	1.61 (+27%)	1.24 (−3%)	1.14 (−10%)
No-Control Area	1.26	1.39 (+11%)	1.26 (0%)	1.36 (+8%)

(B) after	No badger control	Badger culling	Badger vaccination	Badger culling & ring vaccination
Core plus Ring	1.30	0.69 (−47%)	0.92 (−29%)	0.41 (−68%)
Core Area	1.33*	0.35 (−74%)	0.66 (−50%)	0.25 (−81%)
Ring Area	1.27	1.02 (−20%)	1.18 (−8%)	0.58 (−55%)
No-Control Area	1.31	1.27 (−3%)	1.26 (−4%)	1.19 (−9%)

(C) whole period	No badger control	Badger culling	Badger vaccination	Badger culling & ring vaccination
Core plus Ring	1.30	0.92 (−29%)	1.01 (−23%)	0.65 (−50%)
Core Area	1.33*	0.53 (−60%)	0.81 (−39%)	0.45 (−66%)
Ring Area	1.27	1.31 (+3%)	1.21 (−5%)	0.86 (−32%)
No-Control Area	1.29	1.33 (+4%)	1.26 (−2%)	1.27 (−1%)

* Note that the numbers of infected badgers per social group are always higher in the core area because control is centred on the highest incidence area.

The percentage change from business as usual is also given. Mean numbers are from 100 runs of the model. Section (A) gives the results during control (years 1–5), (B) after control (years 6–10) and (C) the results over the whole ten year period.

**Figure 1:**
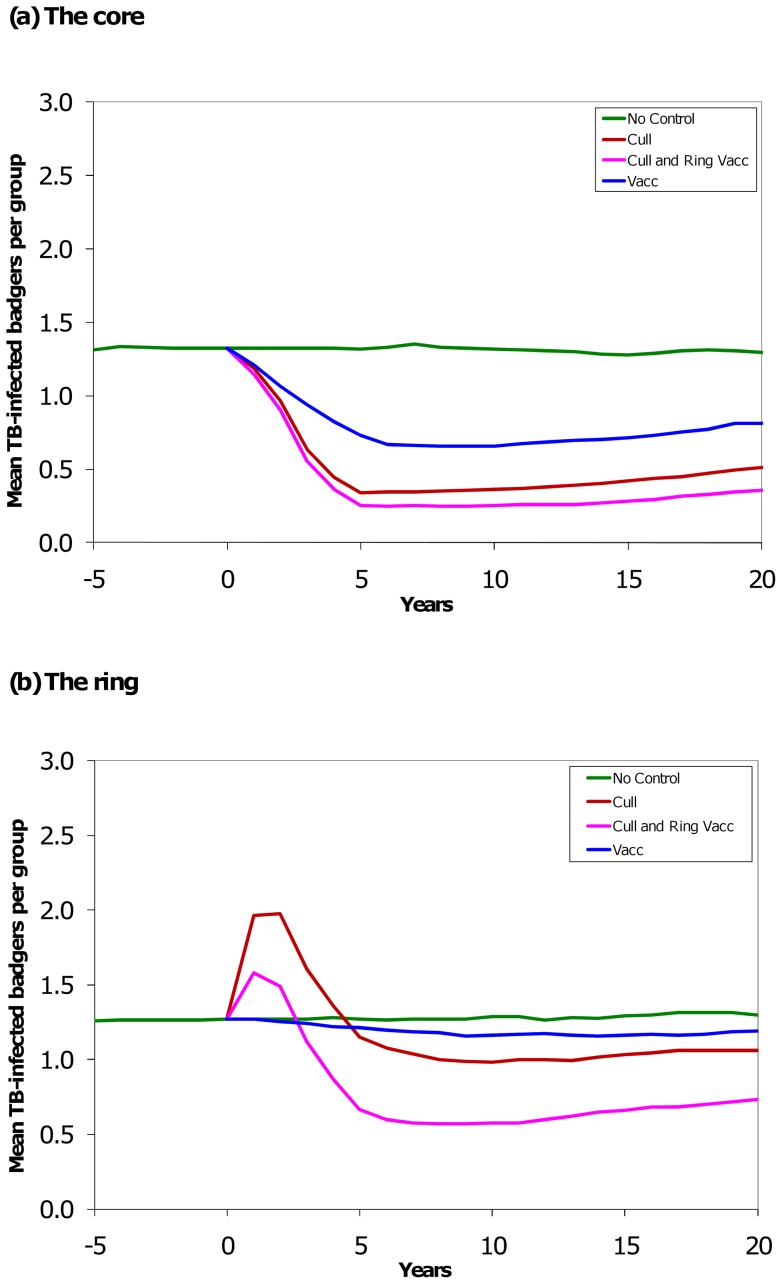
Effect of culling, vaccination, and culling plus ring vaccination on the number of TB-infected badgers. Model outcomes for the mean number of infected badgers per social group at the end of each year. Each control strategy was separately applied in years one to five. For each control strategy the model parameters were re-initialised to match the no-control at year zero. The green line is business as usual, the blue line is vaccination-only in the core area, the brown line is culling-only in the core area, and the pink line is a combination of culling in the core area and vaccination in the adjacent ring area. The combined strategy comprised control over about twice the area as either the culling-only or the vaccination-only strategy and so took about twice the effort.

Badger main setts were distributed randomly over the landscape at a density of 0.75 setts/km^2^, and badger territories were created by tessellation, each grid square assigned to the closest main sett. This approach resulted in a landscape with no geometric bias [17]. Each badger territory was assigned a carrying capacity based on historical field data (maximum number of breeding females) [13] to limit population growth. Cattle grazing land was created by distributing farms at random over the landscape at a density of 0.78 farms/km^2^ and then forming a grazing area within each farm, positioned at random, appropriately sized, and allocated as a beef, dairy or mixed herd. In the model, the badger territories were fully contiguous, whereas only some cattle grazing areas were in direct contact with a neighbouring herd.

**Table 2:** Effects of culling, vaccination, and culling plus ring vaccination on the mean prevalence of bTB in badgers.

(A) during	No badger control	Badger culling	Badger vaccination	Badger culling & ring vaccination
Core plus Ring	0.17	0.24 (+38%)	0.15 (−16%)	0.19 (+8%)
Core Area	0.18	0.22 (+23%)	0.13 (−28%)	0.19 (+8%)
Ring Area	0.17	0.26 (+54%)	0.16 (−3%)	0.18 (+8%)
No-Control Area	0.17	0.19 (+13%)	0.17 (0%)	0.18 (+10%)

(B) after	No badger control	Badger culling	Badger vaccination	Badger culling & ring vaccination
Core plus Ring	0.17	0.12 (−32%)	0.12 (−30%)	0.07 (−59%)
Core Area	0.18	0.08 (−56%)	0.09 (−51%)	0.05 (−70%)
Ring Area	0.17	0.16 (−6%)	0.16 (−8%)	0.09 (−48%)
No-Control Area	0.17	0.17 (0%)	0.17 (−4%)	0.16 (−7%)

(C) whole period	No badger control	Badger culling	Badger vaccination	Badger culling & ring vaccination
Core plus Ring	0.17	0.18 (+3%)	0.13 (−23%)	0.13 (−26%)
Core Area	0.18	0.15 (−17%)	0.11 (−40%)	0.12 (−31%)
Ring Area	0.17	0.21 (+24%)	0.16 (−5%)	0.14 (−20%)
No-Control Area	0.17	0.18 (+7%)	0.17 (−2%)	0.17 (+1%)

The percentage change from business as usual is also given. Mean prevalence is from 100 runs of the model. Section (A) gives the results during control (years 1–5), (B) after control (years 6–10) and (C) the results over the whole ten year period.

**Figure 2:**
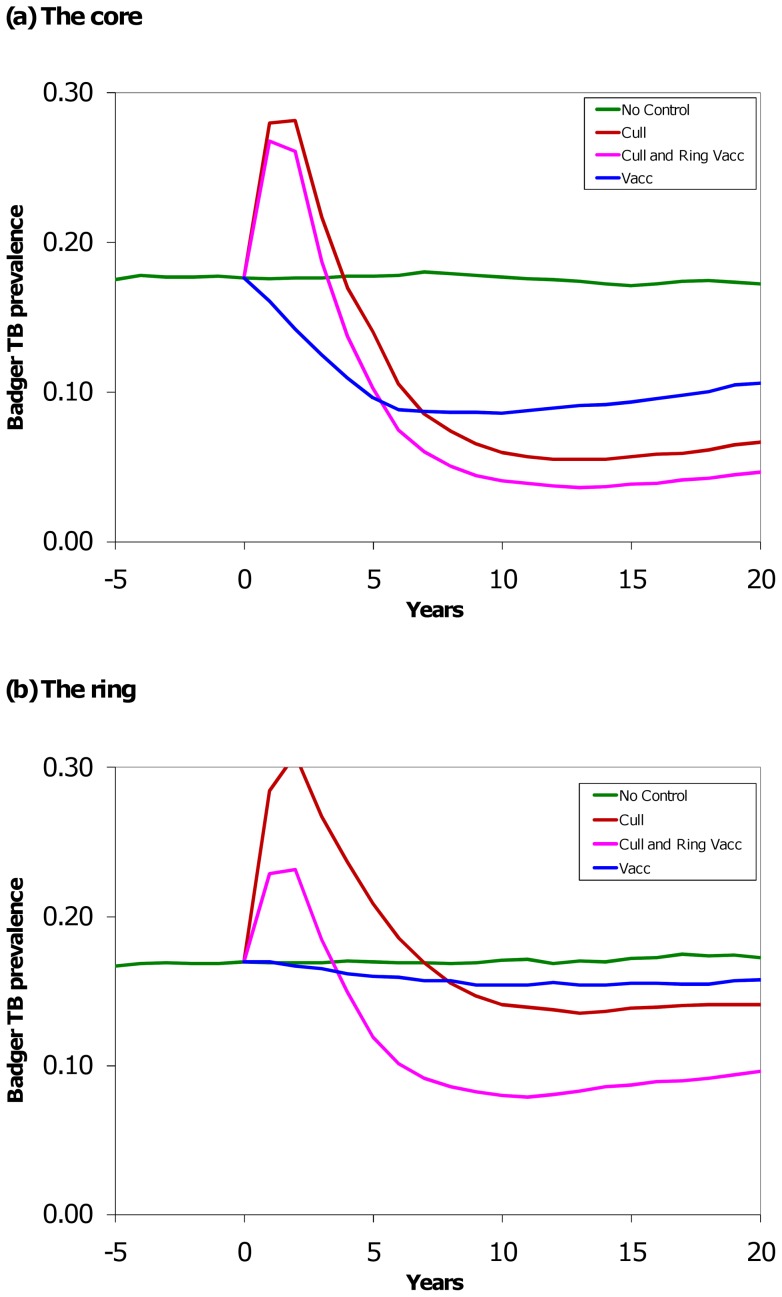
Effect of culling, vaccination, and culling plus ring vaccination on bTB prevalence in badgers. Model outcomes for the badger TB-prevalence for each area at the end of each simulated year. The control strategies were separately applied in years one to five. For each control strategy the model parameters were re-initialised to match the no-control at year zero. The green line is business as usual, the blue line is vaccination-only in the core area, the brown line is culling-only in the core area, and the pink line is a combination of culling in the core area and vaccination in the adjacent ring area. The combined strategy comprised control over about twice the area as either the culling-only or the vaccination-only strategy and so took about twice the effort.

Disease spread was simulated with specified transmission probabilities: within badger group, between badger groups, within cattle herd, between cattle herds, badgers to cattle, and cattle to badgers. Disease transmission was density dependent within each herd or social group in the sense that each possible contact between each individual is given a fixed probability of bTB transmission. The spatial aspect of this model means that local density dependent transmission does not result in global density dependence [15]. A proportion of badgers and cattle were initially infected with bTB at random. A new spatial configuration of territories and farms and new initial populations (badger and cattle) were created for each simulation.

**Table 3:** Effects of culling, vaccination, and culling plus ring vaccination of badgers on disease incidence in cattle reported as the mean annual Cattle Herd Breakdown rate.

(A) during	No badger control	Badger culling	Badger vaccination	Badger culling & ring vaccination
Core plus Ring	0.061	0.056 (−9%)	0.058 (−5%)	0.053 (−14%)
Core Area	0.064	0.048 (−25%)	0.058 (−9%)	0.047 (−27%)
Ring Area	0.059	0.064 (+10%)	0.058 (−1%)	0.059 (0%)
No-Control Area	0.052	0.054 (+3%)	0.051 (−3%)	0.053 (+1%)

(B) after	No badger control	Badger culling	Badger vaccination	Badger culling & ring vaccination
Core plus Ring	0.062	0.046 (−26%)	0.051 (−17%)	0.036 (−42%)
Core Area	0.065	0.037 (−43%)	0.046 (−28%)	0.030 (−53%)
Ring Area	0.060	0.054 (−10%)	0.056 (−6%)	0.042 (−30%)
No-Control Area	0.054	0.050 (−6%)	0.051 (−5%)	0.047 (−12%)

(C) whole period	No badger control	Badger culling	Badger vaccination	Badger culling & ring vaccination
Core plus Ring	0.062	0.051 (−18%)	0.055 (−11%)	0.044 (−28%)
Core Area	0.064	0.042 (−34%)	0.052 (−19%)	0.039 (−40%)
Ring Area	0.059	0.059 (0%)	0.057 (−3%)	0.050 (−15%)
No-Control Area	0.053	0.052 (−2%)	0.051 (−4%)	0.050 (−5%)

The percentage change from business as usual is also given. Mean values are from 100 runs of the model. Section (A) gives the results during control (years 1–5), (B) after control (years 6–10) and (C) the results over the whole ten year period.

**Figure 3:**
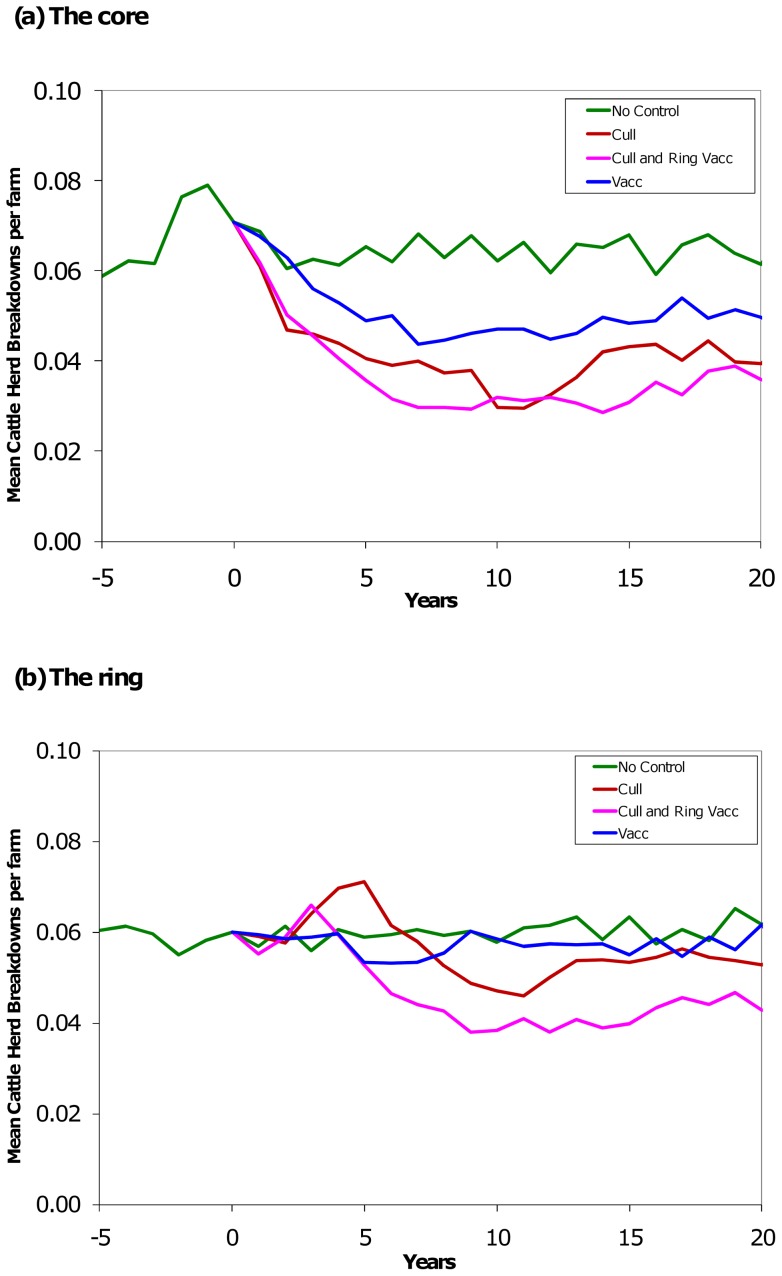
Effect of culling, vaccination, and culling plus ring vaccination on the cattle herd breakdown rate. Model outcomes for the Cattle Herd Breakdown (CHB) rate for each area in terms of the mean number of CHBs per farm per year. The control strategies were separately applied in years one to five. For each control strategy the model parameters were re-initialised to match the no-control at year zero. The green line is business as usual, the blue line is vaccination-only in the core area, the brown line is culling-only in the core area, and the pink line is a combination of culling in the core area and vaccination in the adjacent ring area. The combined strategy comprised control over about twice the area as either the culling-only or the vaccination-only strategy and so took about twice the effort.

### Cattle movements

Cattle movements were simulated so that about 40% of cattle moved each year (on the basis of Cattle Tracing Scheme data – A. Mitchell, Animal Health and Veterinary Laboratories Agency, pers. comm.). Priority was given for young males to move to beef units and females to dairy, and distances moved were minimized wherever possible to simulate market movement patterns. If a herd was too small or too large in terms of its ideal stocking density, extra movements were simulated to redress the balance.

**Table 4:** Effects of culling, vaccination, and culling plus ring vaccination of badgers on the mean total number of Cattle Herd Breakdowns.

(A) during	No badger control	Badger culling	Badger vaccination	Badger culling & ring vaccination
Core plus Ring	71.6	65.5 (−6.1)	67.7 (−3.9)	61.6 (−10.0)
Core Area	37.3	27.9 (−9.4)	33.8 (−3.5)	27.4 (−9.9)
Ring Area	34.4	37.7 (3.3)	34.0 (−0.4)	34.2 (−0.2)
No-Control Area	20.4	21.0 (0.6)	19.8 (−0.6)	20.6 (0.2)

(B) after	No badger control	Badger culling	Badger vaccination	Badger culling & ring vaccination
Core plus Ring	72.6	52.9 (−19.7)	60.0 (−12.6)	42.3 (−30.3)
Core Area	37.8	21.5 (−16.3)	27.1 (−10.7)	17.8 (−20.0)
Ring Area	34.8	31.4 (−3.4)	32.9 (−1.9)	24.5 (−10.3)
No-Control Area	20.9	19.7 (−1.2)	19.8 (−1.1)	18.4 (−2.5)

(C) whole period	No badger control	Badger culling	Badger vaccination	Badger culling & ring vaccination
Core plus Ring	144.2	118.4 (−25.8)	127.7 (−16.5)	103.9 (−40.3)
Core Area	75.0	49.4 (−25.6)	60.9 (−14.1)	45.2 (−29.8)
Ring Area	69.2	69.0 (−0.2)	66.9 (−2.3)	58.8 (−10.4)
No-Control Area	41.3	40.6 (−0.7)	39.6 (−1.7)	39.0 (−2.3)

Figures in parentheses are the differences in the number of breakdowns with respect to business as usual, thus negative numbers are a net reduction in the number of breakdowns. Section (A) gives the results during control (years 1–5), (B) after control (years 6–10) and (C) the results over the whole ten year period.

### Cattle bTB-testing

Cattle were tested routinely at a testing interval determined by the local CHB rate. The testing interval of each simulated parish of approximately 13 km^2^ (one-, two-, three-, or four-yearly) was reassessed annually with the same decision process that is used in real life. The detection of bTB during the testing regime was determined probabilistically to simulate the sensitivity of the bTB skin test, and a positive test triggered slaughter, *post mortem* examination of the reactors, and movement restrictions plus follow-up testing of the contacts.

Premovement testing (PrMT) of cattle was introduced in England in March 2006 to try and limit the spread of undiagnosed bTB into new areas. This required that, in areas already subjected to routine bTB tests every 1 or 2 years, cattle over a certain age had to have a recent negative bTB test before being allowed to move from one farm to another. Premovement testing of cattle was included as a default in the model to simulate the practice used in the field and was applied for long enough that population and disease dynamics had stabilized before badger control was started.

### Variables and inputs

Default parameter values were taken from a previous model version [11] and from available field data (see Text S1). Badger parameters were mostly derived from a single study population (Woodchester Park, southwest England; (see [18], [19], [20], [21]). Badgers were characterized by the following variables: social group, sex, age, and bTB status. The age categories were juvenile, yearling (1 yr old), and adult. The bTB status categories [20] were defined healthy (free of TB infection), infected, infectious, and superinfectious (persistently excreting bTB bacilli). Badger fecundity in the model was density dependent on the basis of a heterogeneous threshold carrying capacity (mean upper threshold of three litters per social group) set at random for each social group [13]. Litter size was modelled probabilistically from a distribution of known litter sizes [22], with a mean of 2.94 cubs/litter and a sex ratio of 1∶1. Mortality rates were taken from Wilkinson *et* *al*. [21]. Badgers were allowed to disperse to smaller social groups, if available, on the basis of sex-dependent probabilities (males more often than females), independent of age and season, and preferentially move to groups with no members of the same sex.

Cattle population parameters (number of dairy and beef farms and stocking densities) were derived from the UK June Census 2004 dataset (Defra, unpubl. data), cattle mortality (slaughter) rates from the Cattle Tracing Scheme (CTS) 2002 to 2004 dataset, and the cattle bTB disease parameters and CHB rates from the UK VetNet dataset. Cattle were characterized by the variables herd, sex, age (30×2-month categories with the last category also used for older cattle), and bTB status (healthy, infected, infectious, superinfectious). Superinfectious cattle were defined as heavily infectious yet anergic (not responding to the bTB skin test) [23]. All female cattle aged over 22 months gave birth to one calf annually (sex ratio 1∶1). Herds were categorized as beef or dairy, and parameter values varied according to herd type. Stocking density distributions from the June Farm Census were dependent on herd type and were used in the model to allocate a stocking density to each farm. Bovine tuberculosis transmission rates for badgers and cattle were adjusted so badgers directly contributed to about 60% of CHBs (when PrMT was in effect), prevalence of bTB in the badger population (before control) stabilized to about 17%, and the mean CHB rate stabilized at about 6% of farms per year when PrMT was in effect [11]. Between-group badger transmission rates were set to one twentieth of the within-group rates to simulate historical spatial and temporal occurrence of diseased social groups [20]. Similarly, between-herd (over-the-fence) rates were set to be one twentieth of the within-herd rates. Within-herd transmission rates for beef herds were set the same as those for dairy. The standard bTB test sensitivity was set at 70% and increased to 90% to simulate the severe interpretation (Defra, unpublished data). Test specificity was set from 99.7 to 99.9%.

### Badger Control Strategies

The model of Wilkinson et al. [11] was modified to allow inclusion of combined badger vaccination and culling strategies, in addition to cull-only and vaccination-only. All simulated badger control was based on intensive large-scale proactive trapping of badgers and either culling, or vaccination by injection and release.

For each simulation, it was assumed that treatment would be initiated in the worst-affected area. Therefore, a bTB-hotspot was located by identifying the parish with the highest CHB rate over the three years prior to initiation of control. A core control area of 150 km^2^ centred around that hot-spot area was then defined (the core area), and a ring of two farm widths (approximately 2 km) around that core area (the ring area) was also defined. The ring area approximated a further 150 km^2^, hence the core area represented just over one-third of the total simulation area (400 km^2^), and the core plus ring areas represented about three-quarters. On average the modelled treatment areas consisted of 117 farms in the core area and 234 farms in the core plus ring. Unless otherwise stated, control lasted 5 years, to approximate the approach of the RBCT, and as suggested by the UK government consultation. The modelled strategies were:

1. Business as usual. No badger control.
2. Badger culling in the core area.
3. Badger vaccination in the core area.
4. Badger culling in the core area plus vaccination in the ring area.

Although these were the treatments considered by the government in England, for completeness of comparison among the approaches, we supplemented these with further simulations with both culling only and vaccination only in a 300 km^2^ area (equivalent to the treatment area under strategy 4), to ensure comparability of effort. Additional simulations were performed with continuous vaccination for 40 years to examine the long-term consequences of this strategy.

It was assumed for the model that 70% of the farms were compliant (accessible for trapping badgers) for either culling or vaccination, in line with reported rates in the RBCT [2]. Badger culling was applied at 70% trapping efficacy [24]. Vaccination was applied at 70% trapping efficacy and a nominal vaccination efficacy of 70%, recognising that recent field trials do not provide full support for this as an absolute measure of efficacy [7]: hence in the model the probability of a susceptible badger in a vaccine control area being trapped, injected with vaccine, and becoming immune to bTB, was 49%.

The model was established and run for 120 simulated years before control started, to obtain stable badger and cattle populations, and patterns of bTB prevalence and distribution, before any control measures were implemented. For clarity most of the pre-control output is not shown on the graphs: the year labelled 1 in the figures represents the first year of control.

### Badger Perturbation

Perturbation of the badger population following culling was included in the model. The precise mechanisms of perturbation that give rise to increased cattle disease are not known, therefore we adopted a pragmatic approach to modelling the process, the outcomes of which are consistent with empirical observations on CHB rate [4], [25]. Here, perturbation was comprised of (a) extra movements of badgers following culling, where badgers would move in to re-colonise badger territories with few or no badgers, and (b) increased badger-to-badger bTB transmission rates in culled areas and nearby territories to simulate higher contact rates that occur as a result of the extra roaming of surviving badgers [26], [27]. In the model, with perturbation, a badger could potentially move two social groups each time step of 2 months. The migration distances simulated by the perturbation routine were thus comparable to those seen in the field after culling [6] [28]. It was assumed that during perturbation, contact between badgers in adjacent territories became similar to those within a territory (i.e. increasing between-group transmission rates to equal within-group rates). The perturbation effect was simulated to extend a width of two badger territories beyond the edge of the culling boundary, which again closely matches the findings during the RBCT.

### Sensitivity Analysis

Previous sensitivity analysis of the model [14] has shown that the modelled badger population was the most sensitive to badger mortality rates, female badger breeding probabilities, and within-group bTB transmission probabilities. Disease prevalence in badgers was most sensitive to badger mortality rates, badger bTB transmission rates, and bTB disease progression rates. The cattle CHB rate was most sensitive to badger mortality rates, and cattle test sensitivity, followed by badger within-group transmission rates and badger bTB disease progression.

Since the output of this model was, in part, used to help inform potential policy development, the sensitivity analysis was designed, not to examine the scale of any changes in magnitude of the outputs, but to determine whether the relative benefits of each badger control strategy were consistent in the face of uncertainty in parameter estimates. Thus, we looked specifically for any parameter changes that altered the order of success of the different strategies, as determined by changing CHB rates.

A novel approach was used for the sensitivity analysis of the model. Parameters were adjusted in a balanced way to maintain badger prevalence and cattle herd breakdown within an appropriate range (see below), whilst analysing the effects on the control strategies that were most pertinent to policy decision-making. Working within this constraint we did not consider any economic analysis of the management, and emphasise that culling plus ring vaccination required approximately twice the effort of the other management strategies, and that vaccination was only applied for the same duration as the other strategies (five years), in line with the government's consultation document.

In this study, rather than a standard one-at-a-time (OAAT) sensitivity analysis, we looked at what the effect of changing parameter values might be on the success (or otherwise) of the choice of control strategies. Therefore, we first ran a standard OAAT sensitivity analysis changing parameters to values considered to be reasonable extreme limits of variability of uncertainty (see Table S1 for the parameter changes). For each of these OAAT changes, the resulting outputs of mean badger prevalence and CHB rate *for the no-control scenario* were examined, and where they had changed too much, one or more other parameters, with the greatest uncertainty, were then also changed to counteract the extreme effect (re-balance the output), and the simulations re-run. The output changes that were considered to be too great, and hence required a re-balance were an increase in badger prevalence greater than 100%, a decrease in badger prevalence of more than 30%, an increase in CHB rate greater than 33%, or a decrease in CHB of more than 17%. The outputs used for this analysis were the means over the 10-year period from the start of control (i.e. 5 years control plus 5 years post-control), so that the size of any changes could be related to the findings reported from the RBCT. The parameters that had to be changed to rebalance the output, and the required sizes of those changes, are listed in Table S2. The only parameter change that was allowed to result in a final output change outside the specified range was the badger-to-badger transmission rates for doubling the badger bTB-prevalence. This was included as it has been suggested that the true badger bTB prevalence might be as much as twice the commonly accepted value, as the standard *post-mortem* tests may be underestimating the numbers of bTB infected badgers [29].

### Outputs

The output parameters calculated at the end of each simulated year were badger population size, badger disease status (number infected and prevalence), and CHB rates. The mean number of bTB-infected badgers per social group is a direct measure of the weight of infection present in wildlife. The mean number of cattle herd breakdowns (CHBs) per farm is a measure of the effect of badger management on the farms, but this measure also includes an assumption on the proportion of herd breakdowns caused by badgers (∼60%) and additional stochastic effects resulting in greater variation. To get a clearer picture of the differences between strategies, we calculated the means for the outputs of two five-year periods: during the five years of control and the subsequent five years. Outputs were summarized across 100 simulations for each strategy. Output metrics were calculated separately for the core area, ring area, and outer no-control area. It was important to include areas outside the culling boundary, so factors such as perturbation, which can affect the overall outcomes, were included in the analysis.

## Results

### Badger Population

For the no-control strategy the 10-year mean badger population was 7.5 badgers per group, of which 1.3 were infected with bTB, giving a prevalence of 17%. As expected, the simulated badger cull had a dramatic effect on the badger population during the years of culling (mean social group size 3.12 in the core during the cull period), and complete recovery of population densities took, on average, ten years after culling ceased. In the core area, the badger population was reduced by about 65% by the end of the 5-year control period. In contrast, badger vaccination caused an insignificant increase in the population in the core.

### bTB-infected badgers

Over the core plus ring area and the whole period of ten years, culling in the core reduced the number of infected badgers by 29%, vaccination by 23% and culling plus ring vaccination by 50% (Table 1). During the five-year period of active control, over the core plus ring area, culling caused a 10% reduction, vaccination a 15% reduction and culling plus ring vaccination a 31% reduction in the number of infected badgers. In the five years after control the benefits of culling exceeded those of vaccination, while culling plus ring vaccination continued to be the best of the three options (Table 1).

Culling caused a marked decrease (maximum mean benefit of −81% at the end of control) in the number of infected badgers in the core (Figure 1a), but an increase (maximum mean increase of +56%) in the ring (Figure 1b) during control. The vaccination-only strategy achieved a decrease in the number of infected badgers in the core (maximum benefit of −50%), and avoided the detrimental effects of perturbation seen with the culling strategies (Figure 1). Culling plus ring vaccination mitigated the detrimental effects of perturbation in the ring outside the cull area resulting in a smaller initial increase in the ring (maximum +28%) (Figure 1b), but overall resulted in a mean decrease of 10% in the ring over the five years of control (Table 1). Overall, the culling plus ring vaccination strategy resulted in the lowest number of infected badgers, both during and after control (Table 1c), in the core (−66%). The next best strategy in the core was culling (−60%), and then vaccination (−39%) (Table 1c). It should also be noted that culling increased the number of infected badgers in the outer no-control area during culling (Table 1a), suggesting that the simulated detrimental effects of culling were not entirely captured in the two-farm widths of the ring.

Since the above strategies differed in the area of control, it is also sensible to compare these three strategies where all the control areas are approximately 300 km^2^, and thus all require an equivalent effort. Over this larger area of culling, the number of infected badgers decreased by 60% during the five years of control (c.f. 46%), approximately double that of the other strategies (Table S3). Thus, for comparable effort over ten years, culling was the preferred strategy (−75%, although there was a net increase of 10% outside the culled area), then culling with ring vaccination (−51%) and then vaccination (−42%).

### Badger bTB-prevalence

Over the core plus ring area for the period of ten years, culling increased prevalence of bTB in badgers by 3%, vaccination reduced it by 23% and culling plus ring vaccination reduced prevalence by 26% (Table 2). During the five-year period of control, culling increased prevalence by 38%, vaccination reduced it by 16% and culling plus ring vaccination increased prevalence by 8%. In the five years after control, all strategies produced a reduction in prevalence, with culling plus ring vaccination continuing to be the best of the three options (Table 2).

Generally, badger bTB-prevalence followed similar trends to the numbers of infected badgers (Figure 2). However, the two main differences are firstly that the effects of perturbation were more marked (maximum mean increase in prevalence was 95% in the ring), and secondly that those effects were clearly shown not only for the whole area, but also in the core (Figure 2a). However, this latter increase in prevalence, when off set against a reduction in badger density by culling was not sufficient to cause an increase in the final *number* of infected badgers.

Again, when comparing these strategies with a similar amount of effort (300 km^2^), badger culling reduced prevalence by 34% over ten years (although note that outside this larger culled area prevalence increased by 25%). Vaccination gave a 43% reduction in prevalence with no significant edge effect, and culling plus ring vaccination reduced prevalence by 24% (Table S4).

### Cattle herd breakdowns

The 10-year mean rate of cattle herd breakdowns with business as usual was approximately 6 per year per 100 farms. Over the core plus ring area and the whole period of ten years, badger culling reduced the herd breakdown rate by 18%, vaccination by 11% and culling plus ring vaccination strategy reduced the CHB rate by 28% (Table 3). During the five-year period of control, culling caused an 9% reduction, vaccination a 5% reduction and culling plus ring vaccination a 14% reduction in CHB rate. In the five years after control this order of preference stayed the same (Table 3).

The CHB rate followed the trends of the numbers of infected badgers, although with more variation (Figure 3). Despite this stochasticity, it can be seen that culling plus ring vaccination was more successful than the cull-only strategy, which in turn was more successful than the vaccination-only strategy in both the core (Figure 3a) and the ring (Figure 3b), although these differences were less pronounced for CHBs than for infected badgers.

Thus across the core plus ring area, when compared to business as usual (mean 144.2 breakdowns over 10 years), the best badger control strategy over ten years was culling plus ring vaccination (mean 103.9 breakdowns over ten years), then culling (mean 118.4) and then vaccination (mean 127.7) (Table 4). Thus, over a ten-year period, vaccination in the core prevented 16.5 breakdowns, culling in the core prevented 25.8 and culling plus ring vaccination prevented 40.3.

When the control areas were comparable in area (300 km^2^) and effort, compared to business as usual (147 breakdowns over 10 years), badger culling was the preferred strategy (mean 79.6 breakdowns), then culling plus ring vaccination (mean 102.3) and then vaccination (mean 115.3) (Tables S5 and S6).

### Sensitivity Analysis

There was a clear preference for the culling plus ring vaccination strategy among the original three choices. Sensitivity analysis was performed to determine if changes to any of the model's parameters would lead us to change this decision. Adjusting nine parameter values to plausible extremes resulted in either badger prevalence or cattle herd breakdowns changing to be outside the values observed in reality. To compensate for this the most highly uncertain parameters (i.e. disease transmission rates) were adjusted to bring the model output into line with field data (Table S2). Additionally, we changed the badger-to-badger transmission rates (intentionally increased to double disease prevalence in badgers to compensate for under-reporting with the currently available diagnostic tests) and then adjusted interspecies transmission to match disease incidence in cattle.

In the absence of control, prevalence of bTB in badgers was most sensitive to changes in bTB progression in badgers, badger carrying capacity, and mortality, particularly pre-emergent mortality (Table S7). It can be seen from this table that changes in the order of a few percent are due to chance (e.g. reducing vaccine sero-conversion was associated with a 3% reduction in prevalence even without control being applied). The parameter changes that had most *further* effect on the badger prevalence because control was being implemented (i.e. in addition to the sensitivity shown for the no-control strategy) were duration of perturbation, trapping efficacy, land-owner compliance, and increasing badger-to-badger transmission rates to double the prevalence.

In the absence of badger control, the CHB rate was most sensitive to changes in cattle stocking density, farm density, bTB progression in cattle, and bTB progression in badgers (Table S8). The parameter changes that had most *further* effect on the CHB rate because control was being implemented (i.e. in addition to the sensitivity shown for the no-control strategy) were perturbation period, badger-to-cattle bTB transmission rates, badger trapping efficacy, and land-owner compliance.

However, in order to assess the outcome of uncertainty on the choice of strategy we need to compare each strategy against each other, in this case using no control as the default position (Table S9). With the default parameter settings, culling plus ring vaccination strategy was most successful at reducing the CHB rate (−21% over the entire simulated area), culling-only was the next best strategy (−13%), followed by vaccination-only (−9%). Despite all the parameter changes, the ranking of the culling plus ring vaccination strategy did not change.

When comparing culling-only and vaccination-only, vaccination became the preferred strategy with any of the following: decreased landowner compliance, decreased cull efficacy, increased duration of perturbation, and decreased number of badger groups (Table S9).

Given that the parameters that most affected the choice of strategy included two management variables, we needed to investigate further. If, for example, badger culling efficacy reduced from the currently proposed minimum of 70%, then one recourse might be to suspend the badger control, which could potentially make herd breakdown rates worse. Thus we ran additional sensitivity analyses to investigate the effect of stopping control early, and of stopping control early because of either low rates of land access for badger management (drop-out or reduced compliance) or low trapping/culling efficacy (in case of deliberate disruption or less effective delivery of culling, e.g. reliance on ineffective methods or poor co-ordination).

The model showed an increase in CHBs when culling was suspended early. This was the case when the culling-only strategy was stopped after just one year. In addition, the model confirms the idea that culling needs to continue for a minimum of four years to gain an overall benefit in CHB rate (Figure S1). The adverse effects were less pronounced for the combined cull and vaccination strategy, and the vaccination-only strategy did not show any adverse effects of early stopping. The adverse effects of stopping culling early were further exacerbated if either the compliance or the trapping efficacy were lower than expected (Figures S2 and S3). These model results strongly suggest that if either compliance or trapping efficacy were as low as 50%, then overall reductions in CHB numbers would be unlikely to be realized at all with a cull-only strategy of five years or less.

It can be argued that a vaccination strategy should not run for just five years, so additional sensitivity analysis was performed by running all control strategies continuously for 40 years. The output of these simulations indicates that (although not proposed as a strategy) continuous culling leads to the greatest reduction in the number of infected badgers, badger prevalence and herd breakdown rate. Culling with ring vaccination in the next best strategy, and then vaccination on its own (Tables S10, S11, S12, S13).

## Discussion

In this study we examined the effects of proposed badger management strategies on the epidemiology of bTB in badgers and cattle. Sensitivity analysis of the model has identified that cattle herd breakdowns (CHBs) were most sensitive to cattle stocking density, cattle farm density, and TB progression in both cattle and badgers. This agrees with findings that increasing herd size is associated with a greater risk of a breakdown [30], [31]. The approach used here for sensitivity analysis could usefully be adopted for many models that are used to inform decision-making. It is not the size of the change in the output value that is important, but rather whether any natural variability, or uncertainty, in parameter values, would lead us to make a different management decision. We believe this approach of adjusting and rebalancing uncertain parameters (to match output with field data) is a useful method to examine model sensitivity for management decisions.

How does the model compare to field data? The specifics of the RBCT field trial differed from the simulation model in having variable start dates to each treatment area and a break in trapping during one year (2001). Thus, it is hard to identify the exact same periods of time (e.g. the post-cull period in the RBCT was analysed from 12 months after culling ceased, whereas in the simulation we used the month of cessation). Nevertheless, the results are remarkably similar. Badger culling in the RBCT caused a reduction in CHB of 23% (95% CI = −32% to −12%) in the core during the trial and by 38% (95%CI = −48% to −25%) in the period after the trial [4], compared to the modelled mean estimates of −25% and −43% respectively. In the adjoining ring the RBCT data indicate a 24% increase (95%CI = −1% to 56%) during the trial and a 5% decrease (95% CI = −31% to 30%) after the trial, compared to means of +10% and −10% respectively in the simulation. Thus, despite the differences in methodology, no qualitative difference occurred in the herd incident rate. Additionally, after 5 years of trapping the model predicted a 65% reduction in badger density which is very similar to the approximately 70% seen in the RBCT [32]. Prevalence of bTB in badgers at the end of the RBCT had increased by a factor 1.92 [33], although this figures includes a significant increase caused by the suspension of cattle testing in 2001. In the core area average prevalence in the badger increased by a maximum factor of 1.68, and in the ring by 1.95 (Figure 2).

It is important to note that we were not trying to optimise badger management in terms of efficacy, nor cost effectiveness, but to compare between three proposed management strategies that emerged from Defra's public consultation on badger culling in England. It is important to note that the proposed control strategies tested in this modelling study were not equal in either the area managed or the effort deployed, so comparisons between the proposed strategies must be made in context and with caution, and are not predictions for specific geographical areas. Although the combined culling plus ring vaccination appeared better than either culling-only or vaccination-only, the area over which control was applied, and therefore the effort deployed, was about twice as large for this combined strategy as for the other two. So it would be incorrect to assume that a combined strategy is better *per-se* than either single strategy. Indeed, for comparable areas of control, culling appears to be the best strategy (see Table S8), although this makes no comment on its public or economic acceptability.

Vaccination of badgers is an optional or additional approach to disease control because there is now a licensed vaccine, BadgerBCG. Our modelling has shown that while the differences between the outcomes of strategies using culling and/or vaccinating badgers are quite modest (∼17–41 CHBs prevented over 10 years out of an expected 144: Table 4), their risk profile is markedly different. Culling results in the known hazard of perturbation, leading to increased CHBs in the periphery of the culling area. Culling also risks being ineffective or making the disease situation worse, if it is conducted partially (because of low compliance) or ineffectually (because of disruption or poor co-ordination) or it is stopped early (because of licensing infractions or changes in policy). Vaccination carries no comparable risks or hazards. However, as a management strategy in its own right, it would not be used for just five years, as previous work suggests that bTB elimination using badger vaccine would take many years, and clearly depends on vaccine efficacy [10], [34].

Another important caveat is that because there is marked stochasticity in the simulated cattle herd breakdown rates, despite averaging over 100 simulations, there is no guarantee that real life will follow the mean result. As such the model results should be treated as a guide to the relative likelihood of outcomes from the control strategies tested and compared, rather than a prediction. Similar to the stochasticity in the model, there are sometimes confounding factors in the real world (not included in the model) that can lead to unexpected results. Specifically, the model assumed that the background rate of herd breakdowns, farm size, herd size and cattle management do not change over the ten years. Over a 300 km^2^ area, over a ten-year period, vaccination in the core prevented 16.5 breakdowns, culling in the core prevented 25.8 and culling plus ring vaccination prevented 40.3. Thus the difference between strategies with comparable effort (culling and vaccination) appears to be less than one herd breakdown per year. Thus, in the real world, the difference between these strategies would be difficult to determine statistically in any but the longest data sets.

For many parameters, uncertainty will affect the outcome of badger management in similar ways, thus retaining the order of preference of the three strategies tested. However, for two badger parameters this order of preference changed. Badger perturbation was simulated as an increase in movement rates and disease transmission for a 12-month period after culling starts. This period resulted in CHB rates most similar to those seen during and after the RBCT (see [4]). However, the form and duration of badger social perturbation is still poorly understood and significant changes to our assumption may alter the order of preference. The badger perturbation effect seen in the model output was most striking in terms of the badger prevalence (with an increase up to 95%), rather than the number of infected badgers (compare Figures 1 and 2). Assuming the pressure of disease transmission from badgers to cattle is correlated more closely with actual numbers of bTB-infected badgers rather than prevalence, we expect that the perturbation effect would be partly mitigated if sufficient badgers were removed, as this model has suggested and the RBCT has demonstrated [3], [19]. We therefore have to be very cautious in interpreting the consequences of a change in disease prevalence in a culled badger population.

Culling plus ring vaccination did mitigate the effect of perturbation to some extent. A major concern of applying a culling strategy has been that farms adjacent to the control area could suffer an increase in herd breakdowns. This study suggests that such an undesirable effect could be somewhat reduced by applying a ring of badger vaccination. However, if cull efficacy, or land access (compliance) rates are much below the anticipated 70%, then no overall benefit from either approach that included culling, may be seen even after five years of control.

## Supporting Information

Figure S1 For each area, the mean Cattle Herd Breakdown (CHB) rate was calculated for years one to ten, for a range of control durations of between one and eight years, and for the different control strategies. The green line (circles) is no-control, the blue line (diamonds) is vaccination-only in the core area, the brown line (triangles) is culling-only in the core area, and the pink line (squares) is a combination of culling in the core area, and vaccination in the adjacent ring area. Generally, the shorter the duration of control, the less was the success of the control compared with the no-control strategy. The combined strategy comprised control over about twice the area as either the culling-only or the vaccination-only strategy.
(DOC)

Figure S2 For each area, the mean Cattle Herd Breakdown (CHB) rate was calculated for years one to ten, for a range of control durations of between one and five years, and for the different control strategies. In addition the compliance for all control areas was set to a low value of 50% of farms. The green line (circles) is no-control, the blue line (diamonds) is vaccination-only in the core area, the brown line (triangles) is culling-only in the core area, and the pink line (squares) is a combination of culling in the core area, and vaccination in the adjacent ring area. Generally, the shorter the duration of control, the less was the success of the control compared with the no-control strategy. The combined strategy comprised control over about twice the area as either the culling-only or the vaccination-only strategy.
(DOC)

Figure S3 For each area, the mean Cattle Herd Breakdown (CHB) rate was calculated for years one to ten, for a range of control durations of between one and five years, and for the different control strategies. In addition the badger trapping efficacy for all control was set to a low value of 50% probability. The green line (circles) is no-control, the blue line (diamonds) is vaccination-only in the core area, the brown line (triangles) is culling-only in the core area, and the pink line (squares) is a combination of culling in the core area, and vaccination in the adjacent ring area. Generally, the shorter the duration of control, the less was the success of the control compared with the no-control strategy. The combined strategy comprised control over about twice the area as either the culling-only or the vaccination-only strategy.
(DOC)

Table S1 Sensitivity Analysis: the parameters and their percentage changes used in the sensitivity analysis.
(DOC)

Table S2 Sensitivity Analysis: additional parameter changes required to re-balance the outputs to within defined limits.
(DOC)

Table S3 Effects of culling, vaccination, and culling plus ring vaccination on the number of infected badgers, per social group, for the different areas of the grid, over each five-year period, for a control area of 300 km^2^.
(DOC)

Table S4 Effects of culling, vaccination, and culling plus ring vaccination on the prevalence of bTB in badgers for the different areas of the grid, over each five-year period, for a control area of 300 km^2^.
(DOC)

Table S5 Effects of culling, vaccination, and culling plus ring vaccination on the mean Cattle Herd Breakdown rate for the different areas of the grid, over each five-year period, for a control area of 300 km^2^.
(DOC)

Table S6 Effects of culling, vaccination, and culling plus ring vaccination of badgers on disease incidence in cattle reported as the mean total number of Cattle Herd Breakdowns occurring in the different areas of the grid, over each five-year period, for a control area of 300 km^2^.
(DOC)

Table S7 Sensitivity Analysis: percentage change in badger TB prevalence (whole grid area) due to parameter changes.
(DOC)

Table S8 Sensitivity Analysis: the percentage change in cattle herd breakdowns per farm (whole grid area) due to parameter changes. The records are ranked on changes in Cattle Herd Breakdown rate of the No-Control strategy.
(DOC)

Table S9 Sensitivity Analysis: the percentage changes in cattle herd breakdowns per farm (whole grid area) due to parameter changes. The records are normalised on changes in Cattle Herd Breakdown rate of the No-Control strategy.
(DOC)

Table S10 Effects of culling, vaccination, and culling plus ring vaccination on the number of infected badgers, per social group, for the different areas of the grid, over each five-year period. Management continues for 40 years.
(DOC)

Table S11 Effects of culling, vaccination, and culling plus ring vaccination on the prevalence of bTB in badgers for the different areas of the grid, over each five-year period. Management continues for 40 years.
(DOC)

Table S12 Effects of culling, vaccination, and culling plus ring vaccination on the mean Cattle Herd Breakdown rate for the different areas of the grid, over each five-year period. Management continues for 40 years.
(DOC)

Table S13 Effects of culling, vaccination, and culling plus ring vaccination of badgers on disease incidence in cattle reported as the mean total number of Cattle Herd Breakdowns occurring in the different areas of the grid, over each five-year period. Figures in parentheses are the differences in the number of breakdowns with respect to business as usual, thus negative numbers are a net reduction in the number of breakdowns. Management continues for 40 years.
(DOC)

Text S1 Default Settings and Parameter Values used in the model.
(DOC)
