# Supplementary material for: Comparing Badger (Meles meles) Management Strategies for Reducing Tuberculosis Incidence in Cattle
Source: PLoS One. 2012 Jun 27;7(6):e39250. doi: 10.1371/journal.pone.0039250 (PMC3384660; doi:10.1371/journal.pone.0039250)
Supplement: Figure S2 — For each area, the mean Cattle Herd Breakdown (CHB) rate was calculated for years one to ten, for a range of control durations of between one and five years, and for the different control strategies. In addition the compliance for all control areas was set to a low value of 50% of farms. The green line (circles) is no-control, the blue line (diamonds) is vaccination-only in the core area, the brown line (triangles) is culling-only in the core area, and the pink line (squares) is a combination of culling in the core area, and vaccination in the adjacent ring area. Generally, the shorter the duration of control, the less was the success of the control compared with the no-control strategy. The combined strategy comprised control over about twice the area as either the culling-only or the vaccination-only strategy. (DOC) [file pone.0039250.s002.doc]

**Figure S2**. Effect of culling, vaccination, and culling plus ring vaccination on the cattle herd breakdown rate (10-year mean), for different control durations, and with a low compliance rate of 50%.


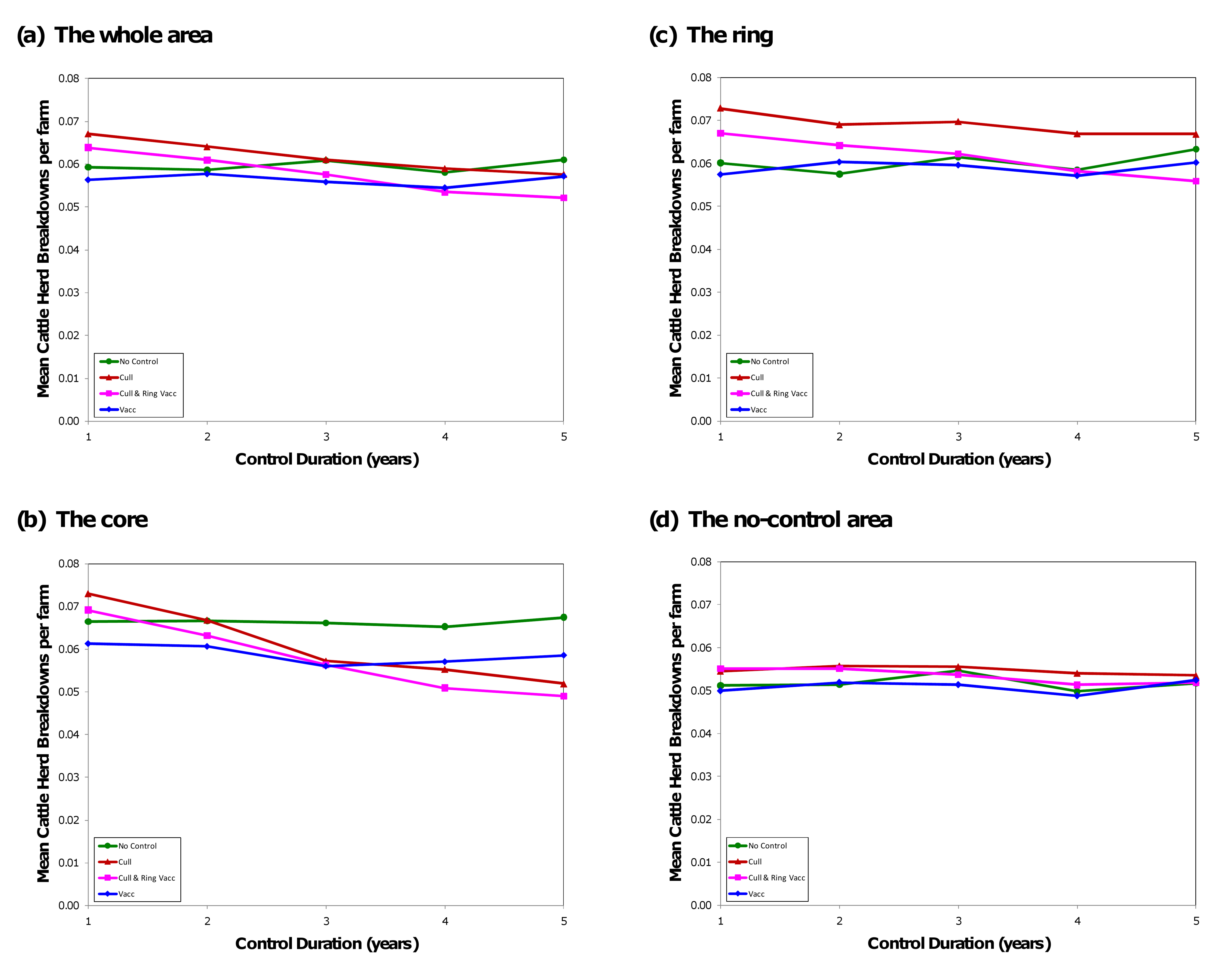


**Figure S2 Legend**

For each area, the mean Cattle Herd Breakdown (CHB) rate was calculated for years one to ten, for a range of control durations of between one and five years, and for the different control strategies. In addition the compliance for all control areas was set to a low value of 50% of farms. The green line (circles) is no-control, the blue line (diamonds) is vaccination-only in the core area, the brown line (triangles) is culling-only in the core area, and the pink line (squares) is a combination of culling in the core area, and vaccination in the adjacent ring area. Generally, the shorter the duration of control, the less was the success of the control compared with the no-control strategy. The combined strategy comprised control over about twice the area as either the culling-only or the vaccination-only strategy.
