# Supplementary material for: Comparing Badger (Meles meles) Management Strategies for Reducing Tuberculosis Incidence in Cattle
Source: PLoS One. 2012 Jun 27;7(6):e39250. doi: 10.1371/journal.pone.0039250 (PMC3384660; doi:10.1371/journal.pone.0039250)
Supplement: Table S1 — Sensitivity Analysis: the parameters and their percentage changes used in the sensitivity analysis. (DOC) [file pone.0039250.s004.doc]

**Table S1**. Sensitivity Analysis: the parameters and their percentage changes used in the sensitivity analysis.

| **Parameter** | **Percent Decrease** | **Percent Increase** |
| --- | --- | --- |
| Badger Groups | -50 | 33 |
| Carrying Capacity | -33 | - |
| Badger Mortality (pre-emergence) | -50 | 50 |
| Badger Mortality (non-super) | -10 | 10 |
| Badger Mortality (super) | -10 | 10 |
| Badger Breeding | -6 | 6 |
| Badger Dispersal (male) | -50 | 50 |
| Badger Dispersal (female) | -50 | 50 |
| Badger TB progression (latent to…) | -50 | 50 |
| Badger TB progression (infectious to latent) | -50 | 50 |
| Badger TB progression (infectious to super) | -50 | 50 |
| Badger TB progression (infected vaccinated) | -50 | - |
| Ba-Ba TB Transmission (2x Prev) | - | 21.5 |
| Ba-Ca TB Transmission | -50 | 50 |
| Compliance | -29 | 29 |
| Perturbation Period | -50 | 50 |
| Trapping efficacy | -29 | 29 |
| Vaccine sero-conversion rate | -29 | 14 |
| Farm Density | -10 | 10 |
| Cattle Stocking density | ~ -20 ^(a)^ | ~ 20 ^(b)^ |
| Cattle TB-test sensitivities | -10 | 10 |
| Cattle Slaughter TB-detect probability | -10 | 10 |
| Cattle TB progression | -50 | 50 |

1. Cattle stocking density was reduced by shifting the distribution curve to the left, and redistributing the lowest value to the 10 next-lowest values.
2. Cattle stocking density was increased by shifting the distribution curve to the right, and redistributing the highest value to the 10 next-highest values
