# Supplementary material for: Comparing Badger (Meles meles) Management Strategies for Reducing Tuberculosis Incidence in Cattle
Source: PLoS One. 2012 Jun 27;7(6):e39250. doi: 10.1371/journal.pone.0039250 (PMC3384660; doi:10.1371/journal.pone.0039250)
Supplement: Table S2 — Sensitivity Analysis: additional parameter changes required to re-balance the outputs to within defined limits. (DOC) [file pone.0039250.s005.doc]

**Table S2**. Sensitivity Analysis: additional parameter changes required to re-balance the outputs to within defined limits. The reference numbers on the left relate to the equivalent numbers in Supporting Info Tables S7 – S9.

| No. | Parameter | Change (%) | 2^nd^ Parameter Changed to Rebalance | 2^nd^ Parameter Change (%) |
| --- | --- | --- | --- | --- |
| 3 | Carrying Capacity | -33 | Badger-TB transmission rates | 20 |
| 4 | Badger Mortality (pre-emergence) | -50 | Badger-TB transmission rates | -13 |
| 5 | Badger Mortality (pre-emergence) | 50 | Badger-TB transmission rates | 18 |
| 6 | Badger Mortality (non-super) | -10 | Badger-TB transmission rates | -15 |
| 7 | Badger Mortality (non-super) | 10 | Badger-TB transmission rates | 20 |
| 8 | Badger Mortality (super) | -10 | Badger-TB transmission rates | -16 |
| 9 | Badger Mortality (super) | 10 | Badger-TB transmission rates | 20 |
| 10 | Badger Breeding | -6 | Badger-TB transmission rates | 10 |
| 11 | Badger Breeding | 6 | Badger-TB transmission rates | -8 |
| 16 | Badger TB progression (latent to…) | -50 | Badger-TB transmission rates | 78 |
| 17 | Badger TB progression (latent to…) | 50 | Badger-TB transmission rates | -20 |
| 18 | Badger TB progression (infectious to latent) | -50 | Badger-TB transmission rates | -5 |
| 19 | Badger TB progression (infectious to latent) | 50 | Badger-TB transmission rates | 5 |
| 20 | Badger TB progression (infectious to super) | -50 | Badger-TB transmission rates | 5 |
| 21 | Badger TB progression (infectious to super) | 50 | Badger-TB transmission rates | -3 |
| 24 | Ba-Ca TB Transmission | -50 | Ca-Ca TB Transmission | 100 |
| 25 | Ba-Ca TB Transmission | 50 | Ca-Ca TB Transmission | -80 |
