# Supplementary material for: Comparing Badger (Meles meles) Management Strategies for Reducing Tuberculosis Incidence in Cattle
Source: PLoS One. 2012 Jun 27;7(6):e39250. doi: 10.1371/journal.pone.0039250 (PMC3384660; doi:10.1371/journal.pone.0039250)
Supplement: Table S3 — Effects of culling, vaccination, and culling plus ring vaccination on the number of infected badgers, per social group, for the different areas of the grid, over each five-year period, for a control area of 300 km2. (DOC) [file pone.0039250.s006.doc]

**Table S3**. Effects of culling, vaccination, and culling plus ring vaccination on the number of infected badgers, per social group, for the different areas of the grid, over each five-year period, for a control area of 300km^2^. Section (A) gives the results during control (years 1-5), (B) after control (years 6-10) and (C) the results over the whole ten year period.

| **(A) during** | **No badger control** | **Badger culling** | **Badger vaccination** | **Badger culling & ring vaccination** |
| --- | --- | --- | --- | --- |
| Control Area | 1.30 | 0.52 (-60%) | 0.91 (-30%) | 0.89 (-32%) |
| No-Control Area | 1.14 | 1.50 (+32%) | 1.13 (-1%) | 1.17 (+2%) |
| **(B) after** | **No badger control** | **Badger culling** | **Badger vaccination** | **Badger culling & ring vaccination** |
| Control Area | 1.32 | 0.14 (-89%) | 0.60 (-54%) | 0.40 (-70%) |
| No-Control Area | 1.06 | 0.93 (-12%) | 1.13 (+6%) | 1.05 (-2%) |
| **(C) whole period** | **No badger control** | **Badger culling** | **Badger vaccination** | **Badger culling & ring vaccination** |
| Control Area | 1.31 | 0.33 (-75%) | 0.76 (-42%) | 0.65 (-51%) |
| No-Control Area | 1.10 | 1.22 (+10%) | 1.13 (+2%) | 1.11 (0%) |
