# Supplementary material for: Comparing Badger (Meles meles) Management Strategies for Reducing Tuberculosis Incidence in Cattle
Source: PLoS One. 2012 Jun 27;7(6):e39250. doi: 10.1371/journal.pone.0039250 (PMC3384660; doi:10.1371/journal.pone.0039250)
Supplement: Table S4 — Effects of culling, vaccination, and culling plus ring vaccination on the prevalence of bTB in badgers for the different areas of the grid, over each five-year period, for a control area of 300 km2. (DOC) [file pone.0039250.s007.doc]

**Table S4**. Effects of culling, vaccination, and culling plus ring vaccination on the prevalence of bTB in badgers for the different areas of the grid, over each five-year period, for a control area of 300km^2^. Section (A) gives the results during control (years 1-5), (B) after control (years 6-10) and (C) the results over the whole ten year period.

| **(A) during** | **No badger control** | **Badger culling** | **Badger vaccination** | **Badger culling & ring vaccination** |
| --- | --- | --- | --- | --- |
| Control Area | 0.17 | 0.19 (+8%) | 0.12 (-30%) | 0.19 (+10%) |
| No-Control Area | 0.17 | 0.25 (+46%) | 0.17 (-1%) | 0.17 (+3%) |
| **(B) after** | **No badger control** | **Badger culling** | **Badger vaccination** | **Badger culling & ring vaccination** |
| Control Area | 0.18 | 0.04 (-75%) | 0.08 (-55%) | 0.07 (-58%) |
| No-Control Area | 0.16 | 0.16 (+3%) | 0.17 (+8%) | 0.15 (-1%) |
| **(C) whole period** | **No badger control** | **Badger culling** | **Badger vaccination** | **Badger culling & ring vaccination** |
| Control Area | 0.17 | 0.12 (-34%) | 0.10 (-43%) | 0.13 (-24%) |
| No-Control Area | 0.16 | 0.20 (+25%) | 0.17 (+3%) | 0.16 (+1%) |
