# Supplementary material for: Comparing Badger (Meles meles) Management Strategies for Reducing Tuberculosis Incidence in Cattle
Source: PLoS One. 2012 Jun 27;7(6):e39250. doi: 10.1371/journal.pone.0039250 (PMC3384660; doi:10.1371/journal.pone.0039250)
Supplement: Table S5 — Effects of culling, vaccination, and culling plus ring vaccination on the mean Cattle Herd Breakdown rate for the different areas of the grid, over each five-year period, for a control area of 300 km2. (DOC) [file pone.0039250.s008.doc]

**Table S5**. Effects of culling, vaccination, and culling plus ring vaccination on the mean Cattle Herd Breakdown rate for the different areas of the grid, over each five-year period, for a control area of 300km^2^. Section (A) gives the results during control (years 1-5), (B) after control (years 6-10) and (C) the results over the whole ten year period.

| **(A) during** | **No badger control** | **Badger culling** | **Badger vaccination** | **Badger culling & ring vaccination** |
| --- | --- | --- | --- | --- |
| Control Area | 0.063 | 0.045 (-29%) | 0.057 (-9%) | 0.053 (-16%) |
| No-Control Area | 0.040 | 0.038 (-5%) | 0.036 (-9%) | 0.037 (-8%) |
| **(B) after** | **No badger control** | **Badger culling** | **Badger vaccination** | **Badger culling & ring vaccination** |
| Control Area | 0.062 | 0.023 (-62%) | 0.041 (-34%) | 0.034 (-45%) |
| No-Control Area | 0.040 | 0.023 (-42%) | 0.031 (-22%) | 0.031 (-24%) |
| **(C) whole period** | **No badger control** | **Badger culling** | **Badger vaccination** | **Badger culling & ring vaccination** |
| Control Area | 0.063 | 0.034 (-46%) | 0.049 (-22%) | 0.044 (-30%) |
| No-Control Area | 0.040 | 0.030 (-24%) | 0.034 (-16%) | 0.034 (-16%) |
