# Supplementary material for: Comparing Badger (Meles meles) Management Strategies for Reducing Tuberculosis Incidence in Cattle
Source: PLoS One. 2012 Jun 27;7(6):e39250. doi: 10.1371/journal.pone.0039250 (PMC3384660; doi:10.1371/journal.pone.0039250)
Supplement: Table S6 — Effects of culling, vaccination, and culling plus ring vaccination of badgers on disease incidence in cattle reported as the mean total number of Cattle Herd Breakdowns occurring in the different areas of the grid, over each five-year period, for a control area of 300 km2. (DOC) [file pone.0039250.s009.doc]

**Table S6**. Effects of culling, vaccination, and culling plus ring vaccination of badgers on disease incidence in cattle reported as the mean total number of Cattle Herd Breakdowns occurring in the different areas of the grid, over each five-year period, for a control area of 300km^2^. Figures in parentheses are the differences in the number of breakdowns with respect to business as usual, thus negative numbers are a net reduction in the number of breakdowns. Section (A) gives the results during control (years 1-5), (B) after control (years 6-10) and (C) the results over the whole ten year period.

| **(A) during** | **No badger control** | **Badger culling** | **Badger vaccination** | **Badger culling & ring vaccination** |
| --- | --- | --- | --- | --- |
| Control Area | 73.9 | 52.2 (-21.7) | 67.1 (-6.8) | 62.2 (-11.7) |
| No-Control Area | 15.6 | 14.7 (-0.9) | 14.1 (-1.5) | 14.4 (-1.2) |
| **(B) after** | **No badger control** | **Badger culling** | **Badger vaccination** | **Badger culling & ring vaccination** |
| Control Area | 73.0 | 27.4 (-45.6) | 48.2 (-24.8) | 40.0 (-33.0) |
| No-Control Area | 15.6 | 9.0 (-6.6) | 12.2 (-3.4) | 11.9 (-3.7) |
| **(C) whole period** | **No badger control** | **Badger culling** | **Badger vaccination** | **Badger culling & ring vaccination** |
| Control Area | 147.0 | 79.6 (-67.4) | 115.3 (-31.7) | 102.3 (-44.7) |
| No-Control Area | 31.2 | 23.8 (-7.4) | 26.3 (-4.9) | 26.3 (-4.9) |
