# Supplementary material for: Comparing Badger (Meles meles) Management Strategies for Reducing Tuberculosis Incidence in Cattle
Source: PLoS One. 2012 Jun 27;7(6):e39250. doi: 10.1371/journal.pone.0039250 (PMC3384660; doi:10.1371/journal.pone.0039250)
Supplement: Table S7 — Sensitivity Analysis: percentage change in badger TB prevalence (whole grid area) due to parameter changes. (DOC) [file pone.0039250.s010.doc]

**Table S7.**

Sensitivity Analysis: percentage change in badger TB prevalence (whole grid area) due to parameter changes (see Supporting Info Tables S1 and S2). The records are ranked on changes in prevalence of the No-Control strategy.

| **No.** | **Parameter** | **%Change** | **No Control** | **Cull** | **Cull & RV** | **Vacc** |
| --- | --- | --- | --- | --- | --- | --- |
| 23 | Ba-Ba TB Transmission (2x Prev) | 21.5 | 101.41 | 87.34 | 93.83 | 106.44 |
| 16 | Badger TB progression (latent to…) | -50 | 41.23 | 37.15 | 42.03 | 46.00 |
| 3 | Carrying Capacity | -33 | 14.41 | 12.23 | 11.08 | 14.86 |
| 20 | Badger TB progression (infectious to super) | -50 | 13.94 | 11.22 | 13.06 | 15.32 |
| 5 | Badger Mortality (pre-emergence) | 50 | 13.18 | 10.54 | 14.29 | 16.21 |
| 7 | Badger Mortality (non-super) | 10 | 11.83 | 10.34 | 11.16 | 13.62 |
| 9 | Badger Mortality (super) | 10 | 10.40 | 10.95 | 8.64 | 11.28 |
| 10 | Breeding | -6 | 8.38 | 7.15 | 9.97 | 9.33 |
| 13 | Dispersal (male) | 50 | 5.13 | 3.63 | 3.26 | 5.91 |
| 1 | Badger Groups | -50 | 5.09 | 10.19 | 11.11 | 3.50 |
| 18 | Badger TB progression (infectious to latent) | -50 | 3.82 | 2.17 | 3.79 | 3.66 |
| 42 | Cattle TB progression | -50 | 3.64 | 3.96 | 2.95 | 5.38 |
| 35 | Farm Density | 10 | 2.94 | 3.79 | 3.18 | 4.60 |
| 19 | Badger TB progression (infectious to latent) | 50 | 2.90 | 4.10 | 3.42 | 5.34 |
| 36 | Cattle Stocking density | -20 | 2.79 | 4.72 | 4.69 | 4.06 |
| 43 | Cattle TB progression | 50 | 2.35 | 0.28 | 1.64 | 1.31 |
| 15 | Dispersal (female) | 50 | 1.91 | 0.05 | 1.86 | 1.22 |
| 22 | Badger TB progression (infected vaccinated) | -50 | 1.72 | 3.27 | 26.91 | 21.51 |
| 34 | Farm Density | -10 | 1.72 | 0.29 | -1.29 | 1.96 |
| 37 | Cattle Stocking density | 20 | 1.45 | 0.30 | 0.98 | 2.56 |
| 33 | Vaccine sero-conversion rate | 14 | 1.25 | 0.46 | -1.44 | 0.14 |
| 29 | Perturbation Period | 50 | 1.23 | 11.82 | 9.25 | 0.78 |
| 30 | Trapping efficacy | -29 | 0.97 | 9.40 | 15.91 | 4.65 |
| 28 | Perturbation Period | -50 | 0.97 | -14.99 | -14.34 | 0.48 |
| 27 | Compliance | 29 | 0.37 | -4.43 | -9.79 | -3.10 |
| 31 | Trapping efficacy | 29 | 0.15 | -6.60 | -8.73 | -1.19 |
| **0** | **Defaults** |  | **0.00** | **0.00** | **0.00** | **0.00** |
| 6 | Badger Mortality (non-super) | -10 | -0.16 | 1.98 | 0.38 | -0.95 |
| 8 | Badger Mortality (super) | -10 | -0.36 | -0.58 | -1.22 | 0.84 |
| 25 | Ba-Ca TB Transmission | 50 | -0.55 | -0.98 | 0.13 | 1.19 |
| 14 | Dispersal (female) | -50 | -0.88 | -3.68 | -3.98 | -1.22 |
| 38 | Cattle TB-test sensitivities | -10 | -1.00 | 0.74 | 0.27 | 2.36 |
| 39 | Cattle TB-test sensitivities | 10 | -1.02 | -0.61 | -1.47 | -0.22 |
| 24 | Ba-Ca TB Transmission | -50 | -1.35 | -0.91 | -1.08 | -0.23 |
| 40 | Cattle Slaughter TB-detect probability | -10 | -1.47 | -2.73 | -0.97 | -2.59 |
| 2 | Badger Groups | 33 | -1.60 | -4.44 | -5.41 | -2.60 |
| 41 | Cattle Slaughter TB-detect probability | 10 | -2.51 | -3.97 | -2.35 | -0.86 |
| 26 | Compliance | -29 | -2.70 | 4.99 | 13.65 | 4.42 |
| 32 | Vaccine sero-conversion rate | -29 | -2.87 | -1.88 | 3.75 | 1.69 |
| 12 | Dispersal (male) | -50 | -3.42 | -3.02 | -3.15 | -3.61 |
| 11 | Breeding | 6 | -4.29 | -5.29 | -7.41 | -3.65 |
| 21 | Badger TB progression (infectious to super) | 50 | -7.50 | -6.31 | -7.18 | -6.04 |
| 4 | Badger Mortality (pre-emergence) | -50 | -7.71 | -7.52 | -7.21 | -8.09 |
| 17 | Badger TB progression (latent to…) | 50 | -8.40 | -5.40 | -9.52 | -9.60 |
