# Supplementary material for: Comparing Badger (Meles meles) Management Strategies for Reducing Tuberculosis Incidence in Cattle
Source: PLoS One. 2012 Jun 27;7(6):e39250. doi: 10.1371/journal.pone.0039250 (PMC3384660; doi:10.1371/journal.pone.0039250)
Supplement: Table S8 — Sensitivity Analysis: the percentage change in cattle herd breakdowns per farm (whole grid area) due to parameter changes. The records are ranked on changes in Cattle Herd Breakdown rate of the No-Control strategy. (DOC) [file pone.0039250.s011.doc]

**Table S8.**

Sensitivity Analysis: the percentage change in cattle herd breakdowns per farm (whole grid area) due to parameter changes (see Supporting Info Tables S1 and S2). The records are ranked on changes in Cattle Herd Breakdown rate of the No-Control strategy.

| **No.** | **Parameter** | **%Change** | **No Control** | **Cull** | **Cull & RV** | **Vacc** |
| --- | --- | --- | --- | --- | --- | --- |
| 37 | Cattle Stocking density | 20 | 14.72 | 12.70 | 13.96 | 13.33 |
| 34 | Farm Density | -10 | 12.19 | 9.20 | 8.29 | 14.35 |
| 43 | Cattle TB progression | 50 | 9.35 | 8.15 | 4.70 | 7.84 |
| 17 | Badger TB progression (latent to…) | 50 | 9.04 | 12.78 | 8.09 | 9.48 |
| 8 | Badger Mortality (super) | -10 | 7.89 | 8.11 | 5.95 | 8.68 |
| 6 | Badger Mortality (non-super) | -10 | 7.76 | 7.53 | 5.16 | 4.10 |
| 2 | Badger Groups | 33 | 6.89 | 3.06 | 4.04 | 5.19 |
| 7 | Badger Mortality (non-super) | 10 | 5.59 | 2.26 | 1.30 | 4.15 |
| 38 | Cattle TB-test sensitivities | -10 | 5.54 | 7.35 | 8.04 | 8.41 |
| 25 | Ba-Ca TB Transmission | 50 | 5.47 | -1.00 | -4.74 | 1.82 |
| 18 | Badger TB progression (infectious to latent) | -50 | 3.78 | 3.78 | 6.06 | 6.25 |
| 5 | Badger Mortality (pre-emergence) | 50 | 3.73 | 2.02 | 2.74 | 5.06 |
| 4 | Badger Mortality (pre-emergence) | -50 | 3.33 | 3.81 | 1.98 | 3.10 |
| 3 | Carrying Capacity | -33 | 3.31 | 0.65 | -1.71 | 5.32 |
| 13 | Dispersal (male) | 50 | 2.97 | 0.76 | 1.63 | 2.46 |
| 10 | Breeding | -6 | 2.72 | 4.98 | 1.92 | 1.64 |
| 29 | Perturbation Period | 50 | 2.47 | 6.30 | 3.40 | 0.84 |
| 15 | Dispersal (female) | 50 | 2.40 | 2.33 | -0.88 | 3.79 |
| 31 | Trapping efficacy | 29 | 2.29 | -3.24 | -5.84 | 2.70 |
| 20 | Badger TB progression (infectious to super) | -50 | 2.24 | 1.94 | 3.74 | 4.62 |
| 28 | Perturbation Period | -50 | 2.21 | -5.55 | -5.55 | 2.07 |
| 9 | Badger Mortality (super) | 10 | 2.20 | 0.76 | -1.54 | 1.06 |
| 33 | Vaccine sero-conversion rate | 14 | 2.03 | 0.91 | 1.20 | 3.38 |
| 40 | Cattle Slaughter TB-detect probability | -10 | 2.03 | -0.19 | 2.06 | -1.91 |
| 22 | Badger TB progression (infected vaccinated) | -50 | 1.98 | 2.68 | 8.15 | 8.14 |
| 11 | Breeding | 6 | 1.94 | 2.74 | -3.70 | 2.70 |
| 30 | Trapping efficacy | -29 | 1.40 | 11.77 | 13.88 | 3.89 |
| 24 | Ba-Ca TB Transmission | -50 | 1.18 | 4.33 | 5.62 | 3.82 |
| 23 | Ba-Ba TB Transmission (2x Prev) | 21.5 | 0.76 | -1.14 | -3.21 | 4.66 |
| 14 | Dispersal (female) | -50 | 0.67 | -1.17 | -1.19 | 1.15 |
| 12 | Dispersal (male) | -50 | 0.65 | 0.61 | 2.10 | 0.16 |
| 21 | Badger TB progression (infectious to super) | 50 | 0.49 | 0.98 | -0.63 | 4.34 |
| 32 | Vaccine sero-conversion rate | -29 | 0.44 | 2.89 | 2.97 | 3.89 |
| 27 | Compliance | 29 | 0.30 | -4.24 | -9.64 | 1.52 |
| 41 | Cattle Slaughter TB-detect probability | 10 | 0.04 | 0.50 | -2.08 | 1.71 |
| **0** | **Defaults** |  | **0.00** | **0.00** | **0.00** | **0.00** |
| 26 | Compliance | -29 | -1.16 | 10.16 | 11.30 | 3.64 |
| 39 | Cattle TB-test sensitivities | 10 | -1.30 | -2.14 | -3.68 | -1.86 |
| 19 | Badger TB progression (infectious to latent) | 50 | -1.54 | -0.20 | -0.44 | 2.19 |
| 1 | Badger Groups | -50 | -1.97 | 1.02 | -1.80 | -3.31 |
| 16 | Badger TB progression (latent to…) | -50 | -3.02 | -5.75 | -6.23 | -2.04 |
| 35 | Farm Density | 10 | -4.63 | -4.53 | -7.12 | -5.66 |
| 42 | Cattle TB progression | -50 | -5.87 | -7.54 | -8.59 | -6.09 |
| 36 | Cattle Stocking density | -20 | -8.52 | -5.76 | -7.57 | -7.07 |
