# Supplementary material for: Comparing Badger (Meles meles) Management Strategies for Reducing Tuberculosis Incidence in Cattle
Source: PLoS One. 2012 Jun 27;7(6):e39250. doi: 10.1371/journal.pone.0039250 (PMC3384660; doi:10.1371/journal.pone.0039250)
Supplement: Table S9 — Sensitivity Analysis: the percentage changes in cattle herd breakdowns per farm (whole grid area) due to parameter changes. The records are normalised on changes in Cattle Herd Breakdown rate of the No-Control strategy. (DOC) [file pone.0039250.s012.doc]

**Table S9.**

Sensitivity Analysis: the percentage changes in cattle herd breakdowns per farm (whole grid area) due to parameter changes (see Supporting Info Tables S1 and S2). The records are normalised on changes in Cattle Herd Breakdown rate of the No-Control strategy.

| **No.** | **Parameter** | **%Change** | **No Control** | **Cull** | **Cull & RV** | **Vacc** |
| --- | --- | --- | --- | --- | --- | --- |
| 0 | Defaults |  | 0.00 | -12.90 | -21.43 | -9.19 |
| 1 | Badger Groups | -50 | 0.00 | -10.24 | -21.29 | -10.42 |
| 2 | Badger Groups | 33 | 0.00 | -16.02 | -23.53 | -10.63 |
| 3 | Carrying Capacity | -33 | 0.00 | -15.14 | -25.24 | -7.42 |
| 4 | Badger Mortality (pre-emergence) | -50 | 0.00 | -12.50 | -22.46 | -9.39 |
| 5 | Badger Mortality (pre-emergence) | 50 | 0.00 | -14.33 | -22.17 | -8.02 |
| 6 | Badger Mortality (non-super) | -10 | 0.00 | -13.09 | -23.32 | -12.27 |
| 7 | Badger Mortality (non-super) | 10 | 0.00 | -15.65 | -24.62 | -10.42 |
| 8 | Badger Mortality (super) | -10 | 0.00 | -12.73 | -22.84 | -8.52 |
| 9 | Badger Mortality (super) | 10 | 0.00 | -14.13 | -24.31 | -10.20 |
| 10 | Breeding | -6 | 0.00 | -10.99 | -22.04 | -10.14 |
| 11 | Breeding | 6 | 0.00 | -12.21 | -25.77 | -8.51 |
| 12 | Dispersal (male) | -50 | 0.00 | -12.94 | -20.30 | -9.64 |
| 13 | Dispersal (male) | 50 | 0.00 | -14.78 | -22.45 | -9.64 |
| 14 | Dispersal (female) | -50 | 0.00 | -14.49 | -22.88 | -8.76 |
| 15 | Dispersal (female) | 50 | 0.00 | -12.96 | -23.94 | -7.95 |
| 16 | Badger TB progression (latent to…) | -50 | 0.00 | -15.35 | -24.02 | -8.27 |
| 17 | Badger TB progression (latent to…) | 50 | 0.00 | -9.92 | -22.11 | -8.82 |
| 18 | Badger TB progression (infectious to latent) | -50 | 0.00 | -12.90 | -19.70 | -7.03 |
| 19 | Badger TB progression (infectious to latent) | 50 | 0.00 | -11.72 | -20.55 | -5.74 |
| 20 | Badger TB progression (infectious to super) | -50 | 0.00 | -13.16 | -20.28 | -7.08 |
| 21 | Badger TB progression (infectious to super) | 50 | 0.00 | -12.47 | -22.30 | -5.70 |
| 22 | Badger TB progression (infected vaccinated) | -50 | 0.00 | -12.30 | -16.68 | -3.70 |
| 23 | Ba-Ba TB Transmission (2x Prev) | 21.5 | 0.00 | -14.55 | -24.52 | -5.67 |
| 24 | Ba-Ca TB Transmission | -50 | 0.00 | -10.19 | -17.98 | -6.81 |
| 25 | Ba-Ca TB Transmission | 50 | 0.00 | -18.24 | -29.04 | -12.33 |
| 26 | Compliance | -29 | 0.00 | -2.92 | -11.52 | -4.78 |
| 27 | Compliance | 29 | 0.00 | -16.84 | -29.21 | -8.08 |
| 28 | Perturbation Period | -50 | 0.00 | -19.51 | -27.39 | -9.31 |
| 29 | Perturbation Period | 50 | 0.00 | -9.65 | -20.72 | -10.63 |
| 30 | Trapping efficacy | -29 | 0.00 | -4.00 | -11.75 | -6.96 |
| 31 | Trapping efficacy | 29 | 0.00 | -17.61 | -27.67 | -8.82 |
| 32 | Vaccine sero-conversion rate | -29 | 0.00 | -10.78 | -19.45 | -6.06 |
| 33 | Vaccine sero-conversion rate | 14 | 0.00 | -13.85 | -22.06 | -7.99 |
| 34 | Farm Density | -10 | 0.00 | -15.23 | -24.16 | -7.44 |
| 35 | Farm Density | 10 | 0.00 | -12.81 | -23.48 | -10.17 |
| 36 | Cattle Stocking density | -20 | 0.00 | -10.27 | -20.61 | -7.74 |
| 37 | Cattle Stocking density | 20 | 0.00 | -14.44 | -21.95 | -10.29 |
| 38 | Cattle TB-test sensitivities | -10 | 0.00 | -11.41 | -19.57 | -6.71 |
| 39 | Cattle TB-test sensitivities | 10 | 0.00 | -13.64 | -23.32 | -9.70 |
| 40 | Cattle Slaughter TB-detect probability | -10 | 0.00 | -14.80 | -21.40 | -12.69 |
| 41 | Cattle Slaughter TB-detect probability | 10 | 0.00 | -12.50 | -23.10 | -7.68 |
| 42 | Cattle TB progression | -50 | 0.00 | -14.44 | -23.70 | -9.39 |
| 43 | Cattle TB progression | 50 | 0.00 | -13.86 | -24.77 | -10.44 |
