# Supplementary material for: Comparing Badger (Meles meles) Management Strategies for Reducing Tuberculosis Incidence in Cattle
Source: PLoS One. 2012 Jun 27;7(6):e39250. doi: 10.1371/journal.pone.0039250 (PMC3384660; doi:10.1371/journal.pone.0039250)
Supplement: Table S10 — Effects of culling, vaccination, and culling plus ring vaccination on the number of infected badgers, per social group, for the different areas of the grid, over each five-year period. Management continues for 40 years. (DOC) [file pone.0039250.s013.doc]

**Table S10**. Effects of culling, vaccination, and culling plus ring vaccination on the number of infected badgers, per social group, for the different areas of the grid, over each five-year period. Management continues for 40 years. Section (A) gives the results during control (years 1-5), (B) during control (years 6-10), (C) the results over the first ten years and (D) over forty years of control.

| **(A) years 1-5** | **No badger control** | **Badger culling** | **Badger vaccination** | **Badger culling & ring vaccination** |
| --- | --- | --- | --- | --- |
| Control Area | 1.31 | 0.54 (-59%) | 0.92 (-29%) | 0.91 (-30%) |
| No-Control Area | 1.15 | 1.60 (+39%) | 1.20 (+4%) | 1.12 (-3%) |
| **(B) years 6-10** | **No badger control** | **Badger culling** | **Badger vaccination** | **Badger culling & ring vaccination** |
| Control Area | 1.30 | 0.06 (-95%) | 0.48 (-63%) | 0.25 (-81%) |
| No-Control Area | 1.21 | 0.72 (-40%) | 1.21 (0%) | 1.19 (-2%) |
| **(C) over 10 years** | **No badger control** | **Badger culling** | **Badger vaccination** | **Badger culling & ring vaccination** |
| Control Area | 1.30 | 0.30 (-77%) | 0.70 (-46%) | 0.58 (-56%) |
| No-Control Area | 1.18 | 1.16 (-2%) | 1.20 (+2%) | 1.15 (-3%) |
| **(D) over 40 years** | **No badger control** | **Badger culling** | **Badger vaccination** | **Badger culling & ring vaccination** |
| Control Area | 1.32 | 0.08 (-94%) | 0.29 (-78%) | 0.18 (-86) |
| No-Control Area | 1.16 | 0.38 (-67%) | 1.18 (+2%) | 0.95 (-18) |
