# Supplementary material for: Comparing Badger (Meles meles) Management Strategies for Reducing Tuberculosis Incidence in Cattle
Source: PLoS One. 2012 Jun 27;7(6):e39250. doi: 10.1371/journal.pone.0039250 (PMC3384660; doi:10.1371/journal.pone.0039250)
Supplement: Table S11 — Effects of culling, vaccination, and culling plus ring vaccination on the prevalence of bTB in badgers for the different areas of the grid, over each five-year period. Management continues for 40 years. (DOC) [file pone.0039250.s014.doc]

**Table S11**. Effects of culling, vaccination, and culling plus ring vaccination on the prevalence of bTB in badgers for the different areas of the grid, over each five-year period. Management continues for 40 years. Section (A) gives the results during control (years 1-5), (B) during control (years 6-10), (C) the results over the first ten years and (D) over forty years of control.

| **(A) years 1-5** | **No badger control** | **Badger culling** | **Badger vaccination** | **Badger culling & ring vaccination** |
| --- | --- | --- | --- | --- |
| Control Area | 0.17 | 0.19 (+10%) | 0.12 (-30%) | 0.19 (+10%) |
| No-Control Area | 0.18 | 0.28 (+55%) | 0.19 (+3%) | 0.17 (-3%) |
| **(B) years 6-10** | **No badger control** | **Badger culling** | **Badger vaccination** | **Badger culling & ring vaccination** |
| Control Area | 0.17 | 0.05 (-72%) | 0.06 (-64%) | 0.07 (-61%) |
| No-Control Area | 0.19 | 0.16 (-16%) | 0.18 (-2%) | 0.19 (+1%) |
| **(C) over 10 years** | **No badger control** | **Badger culling** | **Badger vaccination** | **Badger culling & ring vaccination** |
| Control Area | 0.17 | 0.12 (-31%) | 0.09 (-47%) | 0.13 (-26%) |
| No-Control Area | 0.18 | 0.22 (+19%) | 0.18 (+1%) | 0.18 (-1%) |
| **(D) over 40 years** | **No badger control** | **Badger culling** | **Badger vaccination** | **Badger culling & ring vaccination** |
| Control Area | 0.18 | 0.03 (-81%) | 0.04 (-78%) | 0.04 (-76) |
| No-Control Area | 0.18 | 0.08 (-58%) | 0.18 (+2%) | 0.15 (-17) |
