# Supplementary material for: Comparing Badger (Meles meles) Management Strategies for Reducing Tuberculosis Incidence in Cattle
Source: PLoS One. 2012 Jun 27;7(6):e39250. doi: 10.1371/journal.pone.0039250 (PMC3384660; doi:10.1371/journal.pone.0039250)
Supplement: Table S12 — Effects of culling, vaccination, and culling plus ring vaccination on the mean Cattle Herd Breakdown rate for the different areas of the grid, over each five-year period. Management continues for 40 years. (DOC) [file pone.0039250.s015.doc]

**Table S12**. Effects of culling, vaccination, and culling plus ring vaccination on the mean Cattle Herd Breakdown rate for the different areas of the grid, over each five-year period. Management continues for 40 years. Section (A) gives the results during control (years 1-5), (B) during control (years 6-10), (C) the results over the first ten years and (D) over forty years of control.

| **(A) years 1-5** | **No badger control** | **Badger culling** | **Badger vaccination** | **Badger culling & ring vaccination** |
| --- | --- | --- | --- | --- |
| Control Area | 0.063 | 0.042 (-32%) | 0.057 (-9%) | 0.053 (-16%) |
| No-Control Area | 0.044 | 0.036 (-17%) | 0.040 (-8%) | 0.039 (-11%) |
| **(B) years 6-10** | **No badger control** | **Badger culling** | **Badger vaccination** | **Badger culling & ring vaccination** |
| Control Area | 0.065 | 0.022 (-66%) | 0.042 (-35%) | 0.034 (-48%) |
| No-Control Area | 0.043 | 0.023 (-46%) | 0.031 (-29%) | 0.029 (-32%) |
| **(C) over 10 years** | **No badger control** | **Badger culling** | **Badger vaccination** | **Badger culling & ring vaccination** |
| Control Area | 0.064 | 0.032 (-50%) | 0.050 (-22%) | 0.043 (-32%) |
| No-Control Area | 0.044 | 0.030 (-32%) | 0.035 (-19%) | 0.034 (-22%) |
| **(D) over 40 years** | **No badger control** | **Badger culling** | **Badger vaccination** | **Badger culling & ring vaccination** |
| Control Area | 0.064 | 0.015 (-76%) | 0.033 (-49%) | 0.027 (-58) |
| No-Control Area | 0.042 | 0.014 (-67%) | 0.029 (-30%) | 0.026 (-38) |
