# Supplementary material for: Comparing Badger (Meles meles) Management Strategies for Reducing Tuberculosis Incidence in Cattle
Source: PLoS One. 2012 Jun 27;7(6):e39250. doi: 10.1371/journal.pone.0039250 (PMC3384660; doi:10.1371/journal.pone.0039250)
Supplement: Table S13 — Effects of culling, vaccination, and culling plus ring vaccination of badgers on disease incidence in cattle reported as the mean total number of Cattle Herd Breakdowns occurring in the different areas of the grid, over each five-year period. Figures in parentheses are the differences in the number of breakdowns with respect to business as usual, thus negative numbers are a net reduction in the number of breakdowns. Management continues for 40 years. (DOC) [file pone.0039250.s016.doc]

**Table S13**. Effects of culling, vaccination, and culling plus ring vaccination of badgers on disease incidence in cattle reported as the mean total number of Cattle Herd Breakdowns occurring in the different areas of the grid, over each five-year period. Figures in parentheses are the differences in the number of breakdowns with respect to business as usual, thus negative numbers are a net reduction in the number of breakdowns. Management continues for 40 years. Section (A) gives the results during control (years 1-5), (B) during control (years 6-10), (C) the results over the first ten years and (D) over forty years of control.

| **(A) years 1-5** | **No badger control** | **Badger culling** | **Badger vaccination** | **Badger culling & ring vaccination** |
| --- | --- | --- | --- | --- |
| Control Area | 73.3 | 49.5 (-23.8) | 66.7 (-6.6) | 61.5 (-11.8) |
| No-Control Area | 17.1 | 14.1 (-3.0) | 15.7 (-1.4) | 15.1 (-2.0) |
| **(B) years 6-10** | **No badger control** | **Badger culling** | **Badger vaccination** | **Badger culling & ring vaccination** |
| Control Area | 75.9 | 25.8 (-50.1) | 49.4 (-26.5) | 39.4 (-36.5) |
| No-Control Area | 16.9 | 9.0 (-7.9) | 12.0 (-4.9) | 11.4 (-5.5) |
| **(C) over 10 years** | **No badger control** | **Badger culling** | **Badger vaccination** | **Badger culling & ring vaccination** |
| Control Area | 149.2 | 75.2 (-74.0) | 116.1 (-33.1) | 100.9 (-48.3) |
| No-Control Area | 34.0 | 23.1 (-10.9) | 27.7 (-6.3) | 26.5 (-7.5) |
| **(D) over 40 years** | **No badger control** | **Badger culling** | **Badger vaccination** | **Badger culling & ring vaccination** |
| Control Area | 595.4 | 144.5 (-450.9) | 305.8 (-289.6) | 248.4 (-347.0) |
| No-Control Area | 131.9 | 43.2 (-88.7) | 92.0 (-39.9) | 81.1 (-50.8) |
