# Supplementary material for: Comparing Badger (Meles meles) Management Strategies for Reducing Tuberculosis Incidence in Cattle
Source: PLoS One. 2012 Jun 27;7(6):e39250. doi: 10.1371/journal.pone.0039250 (PMC3384660; doi:10.1371/journal.pone.0039250)
Supplement: Text S1 — Default Settings and Parameter Values used in the model. (DOC) [file pone.0039250.s017.doc]

Text S1 Parameter Values

## Default Settings and Parameter Values used in the model

Note: probabilities or rates are adjusted for a two-month time step where appropriate

### A.1.1 Temporal Settings:

| Year Badgers added to grid | 1 |
| --- | --- |
| Year cattle added to grid | 20 |
| First Year that routine Test Interval switching is introduced (where parishes can change status from one test-interval type to another if CHB rate low or high). | 50 |
| First year that Pre-Movement Testing (PrMT) is introduced | 100 |
| Years of Badger Management | 120-124 |
| Last Year of each simulation | 160 |
| Number of Simulations | 100 |

### A.1.3 Badger Settings:

| **Initial Badgers Added per social group**  Figures obtained by iterative process to give ratio of badger ages and sexes that the model stabilises at. | | |
| --- | --- | --- |
| Juvenile male | 0 or 1 (mean 0.8) | |
| Yearling male | 0 or 1 (mean 0.6) | |
| Adult male | 1 or 2 (mean 1.2) | |
| Juvenile female | 0 or 1 (mean 0.9) | |
| Yearling female | 0 or 1 (mean 0.7) | |
| Adult female | 2 or 3 (mean 2.1) | |
| **Mortality Rates** Taken from fusion life tables of always-healthy badgers [21] at Woodchester Park. Mortality of first 2-months from pre-capture mortality estimates. Mortality of 2-month periods (including first 2-months immediately after pre-capture) from life table of annual mortality: male = 0.304, female = 0.236. Annual mortality of super-excretors: male = 0.667, female = 0.480.  Note: these probabilities are adjusted to be linearly inversely proportional to group size. | | |
| male 1st 2m pre-emergence | | 0.2400 |
| female 1st 2m pre-emergence | | 0.2400 |
| male not-super-excretor | | 0.0586 |
| female not-super-excretor | | 0.0439 |
| male super-excretor | | 0.1675 |
| female super-excretor | | 0.1033 |
| **Breeding Probabilities** | |  |
| First female | | 0.85 |
| 2nd female [adjustable^(1)^] | | 0.40 +/- |
| 3rd female [adjustable^(1)^] | | 0.40 +/- |
| 4th female [adjustable^(1)^] | | 0.40 +/- |
| ^(1)^ Note: the probabilities of 2nd/3rd/4th female breeding are adjusted to be linearly inversely proportional to group size – so smaller groups may breed back up to size faster. The adjustment is based on the equation: “0.40 + (n - 6.7) * -0.079”, but limited between the values 0.00 and 0.85. | | |

| **Litter Size Probabilities**  Taken from Neal & Cheeseman [22], p160 |  |
| --- | --- |
| 1 cub | 0.08 |
| 2 cubs | 0.18 |
| 3 cubs | 0.51 |
| 4 cubs | 0.18 |
| 5 cubs | 0.05 |
| **Dispersal Probabilities** |  |
| male | 0.009390 |
| female | 0.000834 |
| **Health-Status Transfer Probabilities** |  |
| infected to infectious | 0.0309 |
| infected to super- | 0.0274 |
| male infectious to infected | 0.1660 |
| male infectious to super- | 0.2511 |
| female infectious to infected | 0.1660 |
| female infectious to super- | 0.2511 |
| **Infection Transmission Probabilities**  i=infectious, si=super-infectious  Rates set to give a badger prevalence of ~18%, and a CHB rate of ~ 8% | |
| i-badger-badger within-group | 0.021000 |
| i-badger-badger between-group | 0.001050 |
| i-badger-cow | 0 |
| si-badger-badger within-group | 0.042000 |
| si-badger-badger between-group | 0.002100 |
| si-badger-cow | 0.0005 |

### A.1.4 Cattle Settings:

| **Farm Numbers**  Calculated from June Census 2004. Farms mixed with other species (pigs, sheep etc) divided equally between X1-X4. The values given below are for the 400km2 grid. Numbers are multiplied proportionally for larger grid-sizes to maintain the right farm densities. | |
| --- | --- |
| Total Farms | 312 |
| Beef farms | 70 |
| Dairy farms | 58 |
| Mixed farms | 18 |
| X4 (mixed other sp (mainly cattle)) | 37 |
| X3 farms | 37 |
| X2 | 37 |
| X1 (mixed other sp (mainly others)) | 37 |
| **Grazing Proportions**  Calculated from June Census 2004 |  |
| Beef farms | 0.26 |
| Dairy farms | 0.46 |
| X4 | 0.20 |
| X3 | 0.15 |
| X2 | 0.10 |
| X1 | 0.05 |

| **Stocking Density (Beef) Probabilities**  Calculated from June Census 2004 | |
| --- | --- |
| 0.5 cattle per hectare | 0.093 |
| 1.0 | 0.144 |
| 1.5 | 0.190 |
| 2.0 | 0.187 |
| 2.5 | 0.130 |
| 3.0 | 0.089 |
| 3.5 | 0.054 |
| 4.0 | 0.034 |
| 4.5 | 0.022 |
| 5.0 | 0.016 |
| 5.5 | 0.011 |
| 6.0 | 0.007 |
| 6.5 | 0.005 |
| 7.0 | 0.004 |
| 7.5 | 0.004 |
| 8.0 | 0.002 |
| 8.5 | 0.002 |
| 9.0 | 0.002 |
| 9.5 | 0.002 |
| 10.0 | 0.002 |
| **Stocking Density (Dairy) Probabilities**  Calculated from June Census 2004 | |
| 0.5 cattle per hectare | 0.033 |
| 1.0 | 0.058 |
| 1.5 | 0.115 |
| 2.0 | 0.217 |
| 2.5 | 0.204 |
| 3.0 | 0.151 |
| 3.5 | 0.085 |
| 4.0 | 0.051 |
| 4.5 | 0.028 |
| 5.0 | 0.019 |
| 5.5 | 0.011 |
| 6.0 | 0.010 |
| 6.5 | 0.004 |
| 7.0 | 0.003 |
| 7.5 | 0.001 |
| 8.0 | 0.003 |
| 8.5 | 0.003 |
| 9.0 | 0.000 |
| 9.5 | 0.003 |
| 10.0 | 0.001 |

| **Beef Age/Sex Profile**  Calculated from June Census 2004 | |
| --- | --- |
| male 1-yr-old | 0.16 |
| male 2-yr-old | 0.13 |
| male 3-yr-old | 0.04 |
| male 4-yr-old | 0.00 |
| male 5-yr-old | 0.00 |
| female 1-yr-old | 0.14 |
| female 2-yr-old | 0.18 |
| female 3-yr-old | 0.20 |
| female 4-yr-old | 0.10 |
| female 5-yr-old | 0.05 |
| **Dairy Age/Sex Profile**  Calculated from June Census 2004 | |
| male 1-yr-old | 0.07 |
| male 2-yr-old | 0.05 |
| male 3-yr-old | 0.01 |
| male 4-yr-old | 0.00 |
| male 5-yr-old | 0.00 |
| female 1-yr-old | 0.13 |
| female 2-yr-old | 0.22 |
| female 3-yr-old | 0.30 |
| female 4-yr-old | 0.15 |
| female 5-yr-old | 0.07 |
| Cattle BirthRate (per 2m time step)  Taken from Defra Stats Report (Economics of Milk Production - England and Wales 2002/03, chapter 5: autumn calvers = 0.93 calves per year, spring calvers = 0.96 calves per year) | 0.159 |

| **Mortality Rates**  Calculated from CTS slaughter data 2002-2004 |  |
| --- | --- |
| beef male, 6months x 1 | 0.0186 |
| beef male, 6months x 2 | 0.0113 |
| beef male, 6months x 3 | 0.0821 |
| beef male, 6months x 4 | 0.0698 |
| beef male, 6months x 5 | 0.3958 |
| beef male, 6months x 6 | 0.5479 |
| beef male, 6months x 7 | 0.1642 |
| beef male, 6months x 8 | 0.1796 |
| beef male, 6months x 9 | 0.1573 |
| beef male, 6months x 10 | 0.2028 |
| beef male, 6months x 11+ | 0.1565 |
| beef female, 6months x 1 | 0.0182 |
| beef female, 6months x 2 | 0.0072 |
| beef female, 6months x 3 | 0.0145 |
| beef female, 6months x 4 | 0.1127 |
| beef female, 6months x 5 | 0.3354 |
| beef female, 6months x 6 | 0.2353 |
| beef female, 6months x 7 | 0.1025 |
| beef female, 6months x 8 | 0.1229 |
| beef female, 6months x 9 | 0.1238 |
| beef female, 6months x 10 | 0.1347 |
| beef female, 6months x 11+ | 0.1807 |
| dairy male, 6months x 1 | 0.1394 |
| dairy male, 6months x 2 | 0.0180 |
| dairy male, 6months x 3 | 0.0974 |
| dairy male, 6months x 4 | 0.0594 |
| dairy male, 6months x 5 | 0.3738 |
| dairy male, 6months x 6 | 0.5367 |
| dairy male, 6months x 7 | 0.1691 |
| dairy male, 6months x 8 | 0.1684 |
| dairy male, 6months x 9 | 0.1258 |
| dairy male, 6months x 10 | 0.1616 |
| dairy male, 6months x 11+ | 0.1484 |
| dairy female, 6months x 1 | 0.0565 |
| dairy female, 6months x 2 | 0.0116 |
| dairy female, 6months x 3 | 0.0096 |
| dairy female, 6months x 4 | 0.0303 |
| dairy female, 6months x 5 | 0.0500 |
| dairy female, 6months x 6 | 0.0801 |
| dairy female, 6months x 7 | 0.0804 |
| dairy female, 6months x 8 | 0.0910 |
| dairy female, 6months x 9 | 0.1209 |
| dairy female, 6months x 10 | 0.1328 |
| dairy female, 6months x 11+ | 0.1921 |

| **TB-Test probabilities**  Based on unpublished data analysis from Tony Goodchild | | |
| --- | --- | --- |
| **Standard TB-Test probabilities of Conclusive Reactors** | | |
| health category 1 | 0.0007 | |
| health category 2 | 0.3500 | |
| health category 3 | 0.6900 | |
| health category 4 | 0.0007 | |
| health category 5 | 0.3500 | |
| health category 6 | 0.3500 | |
| **Standard TB-Test probabilities of Inconclusive Reactors** | | |
| health category 1 | 0.0030 | |
| health category 2 | 0.3500 | |
| health category 3 | 0.0100 | |
| health category 4 | 0.0030 | |
| health category 5 | 0.3500 | |
| health category 6 | 0.3500 | |
| **Severe TB-Test probabilities of Conclusive Reactors** | | |
| health category 1 | 0.0020 | |
| health category 2 | 0.4500 | |
| health category 3 | 0.8900 | |
| health category 4 | 0.0020 | |
| health category 5 | 0.4500 | |
| health category 6 | 0.4500 | |
| **Severe TB-Test probabilities of Inconclusive Reactors** | | |
| health category 1 | | 0.0030 |
| health category 2 | | 0.4500 |
| health category 3 | | 0.0100 |
| health category 4 | | 0.0030 |
| health category 5 | | 0.4500 |
| health category 6 | | 0.4500 |
| TB-Detect Probability at Slaughter (of an infected animal)  Calculated from CTS data | | 0.217 |
| **Infection Transmission Probabilities**  Infectious & super-infectious transmission rates not differentiated for cattle. Dairy and Beef are differentiated from Munroe & Dohoo, 1999, Proceedings of the Society for Veterinary Epidemiology and Preventive Medicine Conference. | | |
| dairy-cow to cow within-group | | 0.007100 |
| dairy-cow to cow between-group | | 0.000355 |
| Super-infectious dairy-cow to badger | | 0.000050 |
| Beef-cow to cow within-group | | 0.014300 |
| Beef-cow to cow between-group | | 0.000715 |
| Super-infectious Beef-cow to badger | | 0.000050 |
| **Health-Status Transfer Probs (Disease progression)** from Fischer et al. (2005) Prev. Vet. Med. 67: 283-301. | | |
| male infected to infectious | | 0.42 |
| male infected to super- | | 0.001 |
| female infected to infectious | | 0.42 |
| female infected to super- | | 0.001 |
| male infectious to infected | | 0 |
| male infectious to super- | | 0.001 |
| female infectious to infected | | 0 |
| female infectious to super- | | 0.001 |
